# Integrated Transcriptomic and Metabolomic Analyses Reveal Changes in Aroma- and Taste-Related Substances During the Withering Process of Black Tea

**DOI:** 10.3390/foods13233977

**Published:** 2024-12-09

**Authors:** Bernard Ntezimana, Wenluan Xu, Yuchuan Li, Jingtao Zhou, Sujan Pathak, Yuqiong Chen, Zhi Yu, De Zhang, Dejiang Ni

**Affiliations:** 1 National Key Laboratory for Germplasm Innovation and Utilization for Fruit and Vegetable Horticultural Crops, Wuhan 430070, China; bernardntzmn@gmail.com (B.N.); xwl2808572848@webmail.hzau.edu.cn (W.X.); liyuchuan1118@163.com (Y.L.); zhoujingtao@webmail.hzau.edu.cn (J.Z.); sujanp057@gmail.com (S.P.); yuzhi@mail.hzau.edu.cn (Z.Y.); zdybfq@163.com (D.Z.); 2 College of Horticulture and Forestry Sciences, Huazhong Agricultural University, Wuhan 430070, China

**Keywords:** black tea, withering, transcriptome, metabolomics, volatile compounds, non-volatile compounds

## Abstract

Withering is one of the major processing steps critical for the quality of black tea. In this study, we investigated the mechanisms underlying the physicochemical changes in metabolites and gene expression during the withering process of black tea using metabolomic and transcriptomic approaches, respectively. Based on gas chromatography/mass spectrometry non-targeted metabolomic approaches (GC-MS) and ultra-high performance liquid chromatograph–tandem mass spectrometry (UHPLC-MS/MS), a total of 76 volatile compounds and 160 non-volatile compounds were identified from tea leaves, respectively. RNA-seq analysis revealed that the number of differentially expressed genes (DEGs) for the comparative combination of withering time (i.e., W4h, W6h, W8h, W10h, and W12h) compared with CK (i.e., fresh leaves) were 3634, 2906, 4127, 5736, and 7650, respectively. The core genes in starch metabolism, namely alpha-amylase (AMY) and beta-amylase (BAM), were upregulated as withering time increased. AMY and BAM contributed to the decomposition of starch to increase the soluble sugars. The content of tea leaf alcohols and aldehydes, which are the vital contributors for greenish aroma, gradually decreased as withering time increased due to the downregulation of associated genes while the compounds related to sweet and fruity characteristics increased due to the upregulated expression of related genes. Most DEGs involved in amino acids were significantly upregulated, leading to the increase in free amino acids content. However, DEGs involved in catechins metabolism were generally downregulated during withering, and resulted in a reduction in catechins content and the accumulation of theaflavins. The same trend was observed in alpha-linolenic acid metabolism-related genes that were downregulated and enhanced the reduction in grassy aroma in black tea. The weighted gene co-expression network analysis (WGCNA) of DEGs showed that one module can be associated with more components and one component can be regulated by various modules. Our findings provide new insights into the quality formation of black tea during the withering process.

## 1. Introduction

The beverage of black tea is the most consumed type of tea in the world; it makes up 78% of total consumed tea [1,2]. The higher consumption of black tea is accredited to the enriched important compounds, including taste-related substances, such as flavonoids, theaflavins, amino acids, and soluble sugars, and aroma-related substances, such as terpenoids and α-linolenic acid, which are vital contributors to the quality of final products of black tea [2,3,4]. Tea processing technology greatly affects the content and quality of those chemical components related to tea aroma and taste characteristics [5]. Briefly, black tea is processed in four steps, namely withering, rolling/maceration, fermentation, and drying, and withering plays a vital role [6]. Withering, the first step in the manufacturing of black tea, starts when the tea leaf is detached from the tea plant and ends at the rolling/maceration step. It noteworthy that physicochemical changes during the withering process affect taste and aroma formation by alteration in enzymatic activities to provide a rich flavor and other black tea quality characteristics [7,8]. During the withering process, as the moisture content of fresh leaves decreases from 75% to 60%, some polymers are broken into their monomers [9]. Moreover, this dehydration stress initiates a series of gene expressions in withered tea leaves and consequently affects the formation of tea flavor substances [10,11].

In recent years, researchers have reported a number of factors that can affect the physicochemical properties of tea, including temperature, humidity, withering duration, and air flow rate [12]. Existing studies on the withering process of black tea emphasized dry tea/made tea. For example, previous research reported that the maximum withering degree for black tea was 60%, and at this point, tea obtained the highest sensory quality value with better bioactivity [3]. Another study showed that the high withering temperature of black tea, above 30 °C gradually decreased the quality of the final dry tea and one contributing factor was the unfavorable conditions of indigenous enzymatic activities that lead to the decreased level of theaflavins (TF) and thearubigins (TB); therefore, the study recommended that the most suitable withering temperature was below 30 °C [13]. Theaflavins were reported as one of the most abundant polyphenols responsible for color and taste in black tea; TF is formed by a combination of catechins in the presence of two main enzymes, namely polyphenol oxidase (PPO) and peroxidase (POD) [9]. Our previous research also showed that the optimum withering time of black tea to produce the higher quality of dry tea was 8 h–10 h [6]. Additionally, fresh tea leaves contain higher levels of hexanal. Hexenal and hexenol substances formed through lipid degradation (unsaturated fatty acids) in α-linolenic acid metabolism, which imparted about 90% of the grassy aroma in tea [14]. However, there is limited research on mechanisms underlying the physiological and biochemical changes that occur during the withering process of black tea.

Transcriptome sequencing research has improved the accuracy of monitoring the regulatory effect of DEGs on the formation of tea quality-related compounds during withering, especially after the sequencing of tea plant genomes [15,16,17,18]. The quality of tea depends on the formation of metabolites responsible for a specific function (sweet, bitter, mellow, umami, sour, astringency, and aroma). During the withering process of tea leaves, the cells are still alive and actively express genes, which means that changes in gene expression can impact the formation of tea quality characteristics [19,20]. Therefore, transcriptomic and metabolomic studies on the molecular processes that affect tea quality parameters during withering, can provide a comprehensive understanding on the effects of withering time on the development of black tea quality characteristics, bridging the gap between genotype (gene expression) and phenotype (metabolite composition) [21].

The present research elucidates the effects of different withering time on integrated gene expression and volatile and non-volatile metabolites during black tea process. This research enhances our knowledge of chemical changes necessary to improve black tea processing and to produce black tea of good quality, especially during the withering step.

## 2. Materials and Methods

### 2.1. Chemical Reagents

Catechins, alkaloids, amino acids, organic acids, theaflavins, and other flavonoids standards were purchased at Yuanye Biotechnology company (Shanghai, China). The internal standard (D4-acetaminophen) was bought from Aladdin (Shanghai, China). The Quick RNA Isolation Kit was bought from the Huayueyang Biotechnology company (Beijing, China). The TRUE Script Reverse Transcription (RT) Kit and gDNA Eraser were bought from the Aidlab Biotechnologies company (Beijing, China). The PrimeScript RT Reagent Kit was bought from the Takara Biotechnology company (Dalian, China). The Plant Total Protein Extraction Kit and Bicinchoninic Acid (BCA) Protein Assay Kit were purchased from Boxbio Science & Technology Co., Ltd. (Beijing, China). The other enzyme activity kits were bought from the Boxbio Science & Technology company (Beijing, China). N-alkanes (C3–C25) were bought from O2si (Charleston, SC, USA). The rest of the chemical reagents were of analytical grade.

### 2.2. Preparation of Tea Samples

Fresh tea leaves (*Camellia sinensis* cv. Echa no. 10) with one bud and two leaves were picked from the Experimental Tea Garden of Huazhong Agricultural University (Wuhan, Hubei, China) on 26 May 2022. The withering process was carried out indoors under natural conditions (i.e., 55–65% relative humidity with 25 °C temperature); every 2 h, the leaves were shuffled to redistribute the water content and maintain uniformly withered leaves. A sample of fresh leaves (i.e., CK) was quickly collected before withering and kept in −80 °C, while the other samples were collected at different withering time points: after 4, 6, 8, 10, and 12 h, labeled as W4h, W6h, W8h, W10h, and W12h, respectively, with the following moisture contents: CK (75.22 ± 1.41%), W4h (72.17 ± 1.16%), W6h (70.42 ± 1.12%), W8h (67.76 ± 0.66%), W10h (64.54 ± 0.82%), and W12h (61.09 ± 1.04%). Each sample was directly placed in liquid nitrogen before being taken to an ultra-freezer (−80 °C) to be kept for further analysis.

### 2.3. Extraction, Identification, and Quantification of Non-Volatile Substances in Withered Tea Leaves

Here, we used our previous method [6] with some modification. The tea leaf samples were carefully ground using liquid nitrogen and crushed at 30 Hz for 20 s using a hybrid mill (MM400, Retsch, Germany). Then, 150 mg of powdered samples were weighed and placed in 5 mL centrifuge tubes, then 1.5 mL of pre-cooled 75% methanol solution (containing 7.5 μg/mL of D4-acetaminophen as internal standard) was added, and the mixture was left at 4 °C in the dark for 12 h. Then, after being centrifuged at 4 °C and 8000× *g* for 2 min, the supernatant was filtered through a 0.22 μm filter membrane into a brown injection vial, sealed the parafilm outer seal, and stored at −20 °C for analysis.

Untargeted non-volatile substances were obtained by using ultra-high performance liquid chromatograph–tandem mass spectrometry (UHPLC-MS/MS) (Infinity 1290 series, Agilent, CA, USA), coupled with quadrupole time-of-flight mass spectrometry (Q-TOF/MS; Q-TOF 6520, Agilent, CA, USA) and a chromatographic analysis column of Hypersil Gold (100 × 2.1 mm, 3 μm, Thermo Scientific, Bellefonte, PA, USA). Chromatographic conditions: mobile phase A was 0.1% (*v*/*v*) formic acid in water and mobile phase B was methanol, eluted according to a gradient program, i.e., 0–4 min, 10–15% B; 4–7 min, 15–25% B; 7–9 min, 25–32% B; 9–16 min, 32–40% B; 16–22 min, 40–55% B; and 22–28 min. The injected volume was 3 μL; the mass spectrometry conditions were ESI + mode; the capillary voltage was 3.5 kV; the drying gas temperature and flow rate were 300 °C and 8.0 L/min, respectively; and the spray pressure was 3.5 psi. A non-targeted automatic secondary (Auto MS/MS) scan with three collision energy settings of 10, 20, and 30 V was used. The relative content of the detected compound was calculated as follows:

Relative content (μg/g DW) = Sample peak area/Internal standard peak area × 7.5 × 2/W

where, W is the dry weight of the sample (g).

### 2.4. Extraction, Identification, and Quantification of Volatile Compounds in Withered Tea Leaves

The tea leaves samples were crushed into a powder using liquid nitrogen; then, 1 g of the tea powder was weighed and transferred immediately into a 20 mL vial (Agilent, Palo Alto, CA, USA), and 5 mL of saturated boiled NaCl solution was added to inhibit any enzyme reaction. Then, 100 μL of decanoic acid as internal standard (0.86 μL/100 μL) was added and the vial was closed and tightened by the crimp-top cap and TFE–silicone headspace septa (Agilent), then a solid phase microextraction (SPME) fiber (50/30 µm Divinylbenzene (DVB)/carboxen (CAR)/polydimethylsilioxan (PDMS) Supelco, Bellefonte, PA, USA) was exposed to the sample in the headspace bottle. Next, the bottle was placed in a water bath of 60 °C for 1 h to absorb the volatile organic compounds (VOCs). After sampling, the desorption of the VOCs from the fiber coating was taken in the injection port of the gas chromatography (GC) machine (Model 8890; Agilent) at 230 °C for five minutes in splitless mode. 

The identification and quantification of volatile organic compounds were conducted using an GC machine (Model 8890, Agilent, Waltham, MA, USA) and a mass spectrometer (5977B, Agilent, Shanghai, China) with a capillary column of 30 m × 0.25 mm × 0.25 µm DB-5MS. Helium (pure) was used as the carrier gas at 1.0 mL/ min (linear flow rate). The temperature of injector was adjusted to 230 °C, while the temperature of the detector was adjusted to 280 °C. The temperature of the oven was adjusted to 40 °C for 3 min, increasing at 45 °C/min to 85 °C, followed by 7 °C/min; to 100 °C at 3 °C/min for 2 min; to 130 °C at 3 °C/min for 2 min; and followed by an increase up to 230 °C at 10 °C/min for 5 min. Mass spectra were obtained in the electron impact (EI) ionization mode at 70 eV. The quadrupole ion source temperature at 230 °C. Mass spectra were scanned in the *m*/*z* range 35–400 amu at 1 s intervals. The identification of VOCs was completed by comparing the mass spectral in the database/library with the retention index (RI, calculated by N-alkanes C3–C25) of National Institute of Standards and Technology (NIST) as used in previous study [3].

### 2.5. Transcriptome Analysis 

The extraction of RNA, the construction of cDNA Library, and the RNA sequencing (RNA-seq) were performed. Total RNA was extracted from the fresh and withered tea samples (i.e., CK/0h, W4h, W6h, W8h, W10h, and W12h), with three biological replicates, using a Quick RNA Extraction Kit (Huayueyang, Beijing, China). The purity of the RNA was measured using ultraviolet spectrophotometry NanoDope (Implen, Westlake Village, CA, USA), and the RNA concentration, RNA Integrity Number (RNI), and the size of RNA fragment was determined by the Bioanalyer 2100 (Agilent, Santa Clara, CA, USA).

The Illumina HiSeq 150 platform was used to build and sequence the cDNA libraries, and 150 bp paired-end reads were eventually produced. The raw sequencing data were processed by trimming the adapters and eliminating the poor-quality reads. The reference genome (*Camellia sinensis* cv. Shuchazao) and gene model annotation files were obtained from tea plant genome database: http://eplant.njau.edu.cn/tea/index.html (accessed on 28 July 2022), which contains a complete shuchazao tea cultivar genome [22]. A tool (Hisat2, version 2.0.5) from Beijing Nuohe Zhiyuan Technology Co., Ltd., Beijing, China. was used to align the paired-end clean reads to the tea plant reference genome [23]. Three biological replicates were performed for all RNA-Seq data.

### 2.6. Functional Annotation of DEGs

Gene expression levels were determined using the number of fragments per kilo-base of transcript per million mapped reads (FPKMs) [24]. The DEGs analysis was used to compare genes in fresh and withered tea leaves. The parameters to identify significant DEGs were set at *p* < 0.001 and | Log2-transformed (fold change) | ≥ 1.5. The DEGs were categorized based on the annotations in Gene Ontology (GO) and the Kyoto Encyclopedia of Genes and Genomes (KEGG). The DEGs were subjected to the GO and KEGG enrichment analysis using the *p* < 0.01 as a level of significant enrichment, and the expression levels of the DEGs were determined using FPKM values.

### 2.7. Validation of DEGs by Quantitative Real-Time PCR (qRT−PCR)

qRT−PCR analysis was used to verify the accuracy of the RNA−Seq results. The qRT-PCR was conducted on the lightCycler 480 II system (Roche Diagnostics GmbH, Mannheim, Germany) using 2× Syrb Green qPCR Mix (TransGen, Beijing, China). The selected DEG primers were designed with primer 5.0 software (PREMIER Biosoft International, Palo Alto, CA, USA). The conditions for PCR were 95 °C for 2 min; 40 cycles of 95 °C for 10 s, 60 °C for 15 s, and 72 °C for 20 s; and 72 °C for 3 min. The relative expression degree of the chosen transcript was normalized against the *Csβ*-actin gene as the internal reference and was determined based on the 2^−ΔΔCt^ algorithm [25]. Each reaction was performed in triplicate based on prepared samples. The DEG primers used for qRT-PCR are presented in Table S3.

### 2.8. Measurement of Key Enzyme Activity During the Withering Process

The activities of the main enzymes in withering process, namely polyphenol oxide (PPO, EC 1.10.3.1), peroxidase (POD, A084,3-1), hydrogen peroxide (H_2_O_2_, A064-1-1), catalase (CAT, A007-1), superoxide anion (O_2_^−^, A052-1-1), superoxide dismutase (SOD, A001-1), protease, α-amylase, β-amylase, and total amylase, were determined using the commercial kits (Boxbio, Beijing, China).

#### 2.8.1. Oxidase Enzymes

All plants have antioxidant activity defense systems, e.g., catalase (CAT) and superoxide dismutase (SOD), against oxidative stress. The SOD (EC 1.15.1.1), as an antioxidant enzyme that catalyzes the conversion of highly unstable superoxide anion radical (O_2_^-^.) into O_2_ along with the less reactive molecule H_2_O_2_ and catalase (EC 1.11.1.6), can catalyze the oxidation of hydrogen peroxide into water.

#### 2.8.2. Reactive Oxygen Species

The main purpose of the defense system is to scavenge the free radicals, especially radical oxygen species (ROS) from the plant cells. The ROS was measured based on the end products using the hydrogen peroxide (H_2_O_2_) and superoxide anion radical (O_2_^−^) kits. The experiment was conducted according to the instructions of the manufacturer, which was bought from Beijing Boxbio Science & Technology Co., Ltd.

#### 2.8.3. Determination of Malondialdehyde (MDA) Content

The end product of lipid peroxidation in a living cell is MDA, which means it can reflect the degree of tissue peroxidation damage. The determination of MDA was performed using commercial kits from Beijing Boxbio Science & Technology Co., Ltd.

### 2.9. Statistical Analysis

The data were analyzed with SPSS13 (IBM, Chicago, IL, USA) software and one-way ANOVA analysis. GraphPad Prism 8.0.2 and Adobe Illustrator CC 2019 were used to generate the graphs. Each experiment was conducted in triplicate and the data were displayed as means ± standard deviation (SD); the post hoc test was carried out using Duncan’s test, and statistical significance was set at *p* < 0.05. The transcriptome analysis was conducted on Beijing Nuohe Zhiyuan Technology Co., Ltd, Beijing China.

## 3. Results and Discussion

The distinctive flavor of tea is the output of the cumulative effect of the constituents present in tea leaves [26]. However, the molecular mechanism of specialized metabolites that provide a unique quality of tea during withering process have not yet been elucidated. Our study showed the effect of different withering times on the taste and aroma (non-volatile and volatile) compounds that contribute to the quality of tea at the molecular level.

### 3.1. Differential Non-Volatile Metabolites During the Withering Process 

In this research, based on ultra-high-performance liquid chromatograph–tandem mass spectrometry (UHPLC-MS/MS, Infinity 1290, Agilent Technologies, Santa Class, CA, USA) coupled with quadrupole time-of-flight mass spectrometry (Q-TOF/MS; Q-TOF, Q-TOF 6520, Q-TOF 6520,Agilent Technologies In., Santa Clara, USA), we identified a total of 68 non-volatile compounds that were differential metabolites (Table S1), including catechins, amino acids, flavonoid glycosides, theaflavins, alkaloids, organic acids, and others.

**Figure 1:**
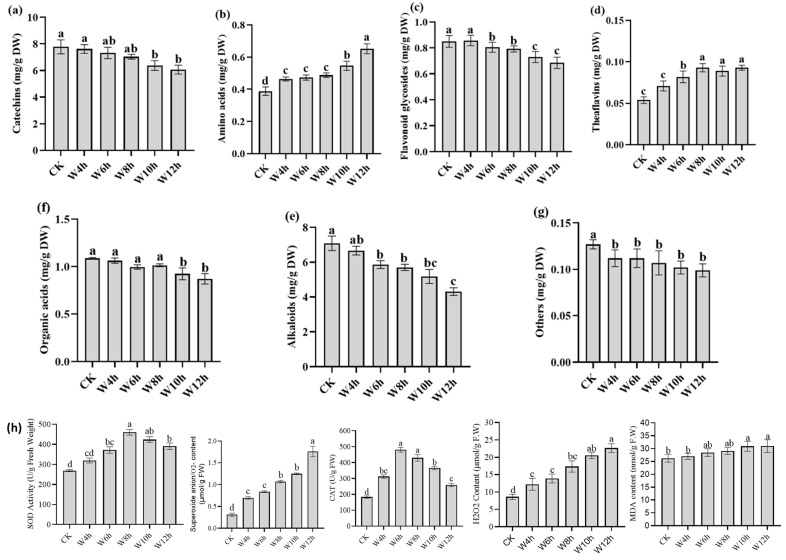
Relative content of the main metabolites under different withering times. (**a**) Catechins, (**b**) amino acids, (**c**) flavonoid glycosides, (**d**) theaflavins, (**e**) alkaloids, (**f**) organic acids, (**g**) others, and (**h**) enzymes activity during withering process. Data are presented as mean ± SD (n = 3), and a significant difference is shown by different letters with a threshold *p* < 0.05, according to Duncan’s test.

In this study, the total catechins content decreased (Figure 1a) with withering time, in the following order: CK (7.85 ± 0.53 mg/g DW) > W4h (7.69 ± 0.33 mg/g DW) > W6h (7.40 ± 0.42 mg/g DW) > W8h (7.15 ± 0.18 mg/g DW) > W10h (6.49 ± 0.36 mg/g DW) > W12h (6.16 ± 0.33 mg/g DW). However, there was no significant difference (*p* > 0.05) between CK and W4h, but the total content kept decreasing (Figure 1a). Epigallocatechin gallate (EGCG), which is the major catechin in tea, gradually reduced from CK (2.309 ± 0.155 mg/g DW) to W12h (1.405 ± 0.081 mg/g DW), and the highest reduction was found between CK and W4h. This reduction in EGCG was associated with formation of theasinensin A content, which increased gradually until W8h and started to decrease afterward (Table S1). A previous study reported that theasinensin A contributes to the bitterness and astringency of tea taste [27]. On the other hand, (+)-catechin (C) content increased gradually as withering time increased from CK (0.176 ± 0.007 mg/g DW) to W12h (0.263 ± 0.006 mg/g DW), although no significant (*p* > 0.05) increase was observed in W8h and W10h treatments. It was suggested that CG hydrolysis reduced CG content while the content of C increased. The catechins contribute to the bitterness and astringent taste in tea [7]. The conversion of catechins to theaflavins in the presence of PPO and POD contribute to the reduction in catechins during tea processing and enhances the palatability of tea as previously reported [9,27]; this might be responsible for the increase in total theaflavins (Figure 1d).

The total amino acids significantly (*p* < 0.05) increased with withering time (Figure 1b) from CK (0.39 ± 0.03 mg/g DW) to W12h (0.65 ± 0.03 mg/g DW). Phenylalanine content gradually increased significantly (*p* < 0.05) from CK (0.087 ± 0.005 mg/g DW) to W12h (0.274 ± 0.011 mg/g DW), with the highest increase in CK and W4h. The same trend was observed in proline content, and this was in agreement with previous research, which reported that phenylalanine and proline are stress response compounds and consequently increased due to withering stress [25]. However, a non-proteinaceous amino acid named theanine is the major amino acid in tea content and was gradually decreased from CK (0.129 ± 0.016 mg/g DW) to W12h (0.079 ± 0.004 mg/g DW) with no significant (*p* > 0.05) difference recorded in W10h and W12h treatments. This reduction might be due to the degradation of theanine into glutamate and ethylamine [6], and glutamate was further converted into glutamine. In this study, there was an increase in arginine, asparagine, and lysine as withering time increased until W12h. Generally, free amino acids contribute to the umami, mellow, and fresh taste of tea infusions [7]

A total of 22 flavonoid glycosides were detected, including kaempferol, myricetin, quercetin, and their glycosides (Table S1). The relative content of flavonoid glycosides gradually reduced with no significant (*p* > 0.05) difference between CK and W4h (Figure 1c), and the total amount reduced from 0.85 ± 0.04 mg/g DW (CK) to 0.68 ± 0.04 mg/g DW (W12h). Previous research showed that the glycoside content gradually reduced during tea processing to increase tea flavor components by breaking the glycosidic bonds in the presence of glycosidase to separate functional groups and sugar [26,28].

The relative content of total alkaloids gradually decreased during the withering process (Figure 1e). Caffeine, a major alkaloid in tea, was reduced from 6.022 ± 0.4 mg/g DW (CK) to (4.219 ± 0.211 mg/g DW (W12h). The decrease in total alkaloid content was due to caffeine’s degradation into imidazoles (1,3-diaza-2,4-cyclopentadiene) [28].

A total of eight organic acids were identified in this study, namely caffeic acid, ferulic acid, gallic acid, p-coumaric acid, chlorogenic acid, d-(-)-quinic acid, salicylic acid, and shikimic acid. Their relative total contents were reduced gradually and insignificantly (*p* > 0.05) from CK to W8h as withering time increased (Figure 1f). The previous research showed that quinic acid increased during withering and reduced during rolling [29]. In tea plants, ferulic acid is the core component of lignin and its content gradually decreased during the withering process. Organic acids are major contributors to the sour taste in tea and are generally amplified during storage [30].

During the withering process, tea leaves experience dehydration stress as the withering time increases [31]. Consequently, this triggered the production of reactive oxygen species (ROS) such as superoxide anion (O_2_^−^), the hydrogen peroxide (H_2_O_2_), and the lipid peroxidation, i.e., malondialdehyde (MDA), as the crucial marker of oxidative stress. However, non-significant (*p* > 0.05) increases in (O_2_^−^) and (H_2_O_2_) levels were recorded in the W4h, W6h, W8h, and W10h treatments (Figure 1h); also, MDA content increased gradually with withering time (Figure 1h). In order to alleviate the oxidative stress effect, more enzymatic antioxidants (superoxide dismutase (SOD), catalase (CAT)) were produced (Figure 1h) by breaking down ROS into H_2_O_2_ in the presence of SOD, then into H_2_O in the presence of CAT [31]. 

### 3.2. Metabolomics Profiling in the Volatile Compounds During Withering

In response to the withering stresses, the tea leaves going through withering process synthesize and release volatile (aroma) compounds as a resistance weapon to the stresses [32], and aroma is an integral part in tea quality. Moreover, the dynamics in the volatile compounds during withering in tea processing is an important step in producing high-quality tea. Volatile compounds such as alcohols, esters, hydrocarbons, ketones, aldehydes, and other organic compounds are responsible for the unique aroma and flavor of the tea [31]. Using GC-MS, a total of 76 volatile compounds were identified as shown in Table S2. Previous research reported that the withering process enriches the contents of alcohols, hydrocarbons, and aldehydes through various chemical reactions, including terpenes biosynthesis, o-methylation, decomposition, and hydrolyzation [31]. Alcohol-related compounds accounted for 79.17% of the total aroma content, followed by esters which accounted for 14.05%; this implies that alcohol has the highest contribution to tea aroma in the withering stage. Among the alcohol compounds, linalool and its oxides, phenylethyl alcohol, dihydrocarveol, and geraniol appeared in abundance compared to others and increased as withering time increased. Trends consistent with the results of the existing study confirmed an increase in alcohol flavor compounds during withering [31]. On the other hand, (Z)-hex-4-en-1-ol, trans-2,4-hexadien-1-ol, 2-ethyl-2-hexen-1-ol, and (E)-6-methylhept-4-en-1-ol, described as leaf alcohol, contributed to the grassy and greenly aroma (unwanted aroma in black tea) [33,34], and gradually decreased as withering time extended (Table S2). In our research, the relative content of aldehyde increased (CK,4.64 ± 0.04 to W12h, 7.86 ± 0.16) as withering time increased; in particular, (2E)-2-octenal increased significantly from CK (1.19 ± 0.07) to W12h (3.33 ± 0.21). The previous study demonstrated that (2E)-2-octenal content increased substantially during withering and the suggested reason was the hydrolysis of glycosides and reaction between amino acids and carbonyl compounds to form strecker aldehydes [35,36]. Moreover, the 2-hexenal content gradually decreased as withering time increased, and 2-hexenal was reported to be an inferior greenish flavor compound in black tea; it was synthesized from lipid degradation during tea processing [33]. The relative content of ester compounds, including methyl salicylate, isopulegyl acetate, neryl formate, hex-3-enyl hexanoate, and cyclohexyl hexanoate, increased progressively as withering time increased (Table S2). Carotenoid-derived volatiles such as β-ionone are a contributor of floral and sweet aroma in black tea and it increased as the withering time was extended (Table S2); this might be a result of the degradation of carotenoid into its monomers [35]. Some volatile compounds were not detected in fresh leaves, such as hexanal, 2-ethyl-, cis-3-nonen-1-ol, trans-7-methyl-3-octene, and n-amylbenzene, and others started to appear in W6h (1,5-dimethyl-1,5-cyclooctadiene, and (Z)-3,7-imethylocta-1,3,6,-triene); at W8h, 2,6-di-tert-butyl-p-benzoquinone and 1-(3,5-di-tert-butyl-4-hydroxy-phenyl)-propan-1-one were detected and increased gradually until W12h (Table S2). This means that, during withering process, there is a time point (about W8h) where various volatile compounds appear. Additionally, previous studies have shown the contribution of amino acids to volatile compound formation through various chemical reactions during the withering process [34,37]. Generally, the withering process enhances the quality of black tea aroma. In this study, the total content of aroma compounds increased from 263.55 ± 15.59 ng/g-dw (CK) to 440.49 ± 15.71 ng/g-dw (12h) (Figure S1).

### 3.3. Transcriptome Results

The transcriptome analysis of fresh and withered leaves samples (W0h, W4h, W6h, W8h, W10h, and W12h) was performed using RNAseq. After RNA sequencing, a total of 121 G cleaned reads (Q30 > 91 and Q20 > 96) with a 0.03% error rate and ~43% GC content, were obtained. The clean reads were mapped to the reference genome (TPIA) with an 86% total mapping rate (4,347,367,415), a 74.825% exon region (495,662,574.7), an 8.528% intron region, and (967,034,911.2) a 16.647% intergenic region. The unmapped clean reads were assembled into 8235 novel genes, with length ranging from 188 to 19,610 bp, and the average length was 1248 bp. A total of 2669 novel genes were annotated in KEGG, and a total of 28,251 novel genes were annotated in GO. The detailed quality of RNA-Seq data and mapping rate to the TPIA genome are shown in Table S4.

#### 3.3.1. The DEGs and Significantly Enriched KEGG Pathways

**Figure 2:**
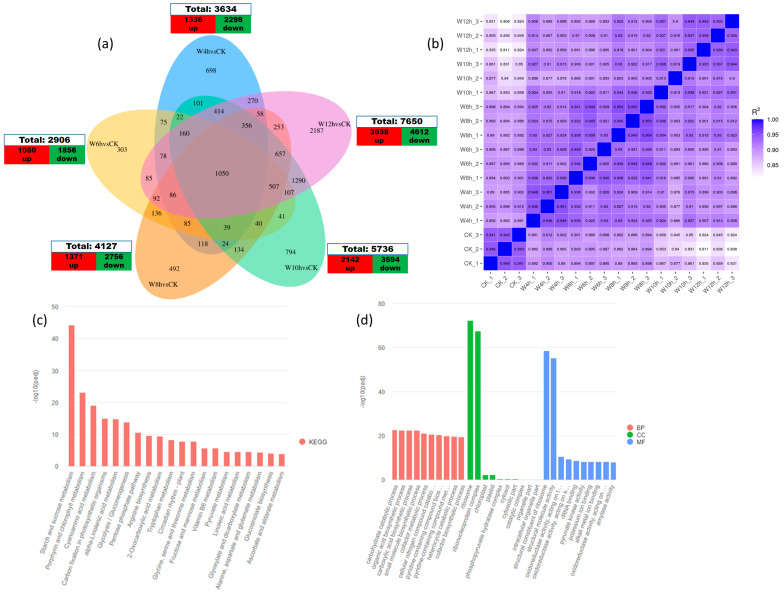
Differentially expressed genes (DEGs) in five groups. (**a**) The identification of DEGs in five groups, where the cross-sectional region shows the shared DEGs. (**b**) Pearson’s correlation between the samples. (**c**) The first 20 significantly enriched pathways (KEGG) in the comparative combination of withering time across all samples. (**d**) Gene Ontology (GO) enrichment analysis across all samples. DEGs were defined using adjusted *p*−value < 0.05 and a |fold-change| > 1.5 as a cut-off.

The numbers of DEGs for the comparative combination of withering time (W4h, W6h, W8h, W10h, and W12h) compared with CK (0h) were 3634, 3006, 4127, 5736, and 7650, respectively. Generally, a total of 8937 genes were upregulated while 15,116 genes were downregulated, as shown in Figure 2a. The differences between the first and second groups might be due to the presence of on- and off-genes shortly after picking the tea leaves. Many genes were turned on for the response of picking stress and some genes were turned off after a while (W6h vs. CK), and genes expression kept increasing gradually. KEGG and GO annotation were used to identify significantly enriched DEGs pathways linked to non-volatile substances, specifically the biosynthesis of catechins, amino acid metabolism, and starch and sucrose metabolisms.

To characterize the DEGs based on GO database, three categories were formed, namely biological process (BP), cellular component (CC), and molecular function (MF). In the BP category, the first three subclasses of DEGs annotations were carbohydrate the catabolic process, the organic acid biosynthetic process, and the carboxylic acid biosynthetic process; in the CC category, the first two subclasses of DEGs were ribosome and the ribonucleoprotein complex, and in the MF category, most of the DEGs were structural components of ribosome and structural molecule activity (Figure 2d). Thus, the tea withering process continues with time and involved different biological activities.

To further analyze the relationships between the enriched DEGs and synthesis of metabolites, we explored the first 20 significantly enriched pathways (KEGG) in the comparative combination of withering time (W4h, W6h, W8h, W10h, and W12h) compared with CK (Figure 2c), in which starch and sucrose metabolism were the foremost significantly enriched pathway metabolisms. These findings showed that the significant changes in gene expression during the withering stage affect the final quality of tea.

The increment of DEGs from 3634 (W4h vs. CK) to 7650 (W12h vs. CK) indicated changes in gene expression during the withering process. The 1050 most common DEGs (Figure 2a), obtained by comparing the five datasets, signified that common DEGs kept functioning biologically until W12h. To comprehend the connection between relative gene expression and metabolite accumulation, this study explored the DEGs in different withering time during black tea processing. The results showed more downregulated DEGs than upregulated DEGs (Figure 2a) and one suggested reason was both the degradation of ribinucleic acids and the reduced synthesis during the withering process [27].

#### 3.3.2. Characterization of the DEGs Involved in Catechin Metabolism During the Withering

**Figure 3:**
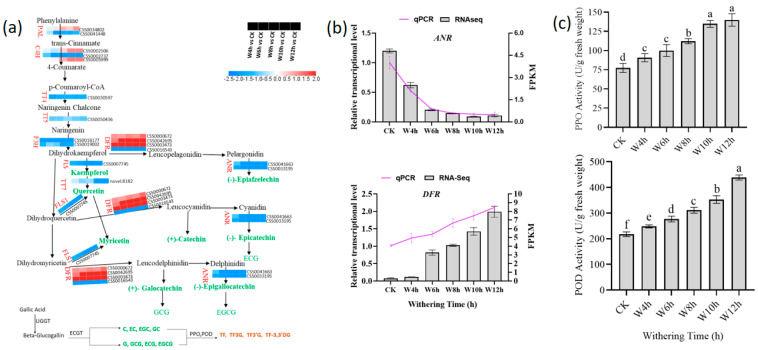
The expression of DEGs involved in catechins metabolism and the activities of oxidation enzymes during the withering process. (**a**) DEGs involved in the biosynthesis of catechins. (**b**) Validation of RNA-seq by qRT-PCR. Column charts and lines represent the RNA-seq values and qRT-PCR results, respectively. (**c**) Activities of PPO and POD. PAL, phenylalanine ammonia-lyase; C4H, cinnamate-4-hydroxylase; F3H, cinnamate-4-hydroxylase; DFR, dihydroflavonol 4-reductase; ANR, anthocyanidin reductase; FLS, flavonol synthase; TT4, chalcone synthase; TT5, chalcone isomerase; TT7, flavonoid 3’-monooxygenase; ECG, (-) epicatechin 3-gallate; GCG, (-) gallocatechin 3- gallate; EGCG, (-) epigallocatechin 3- gallate; (UGGT), UDP-glucose glycoprotein glucosyltransferase; (UCGT) Epicatechin -1-ogalloyl-beta-D-glucose-o-galloyltransferase; TF, theaflavins; TF3G, theaflavin-3-gallate, TF3’G, theaflavin-3’-gallate; TF3,3’DG, theaflavin-3, 3’-digallate; PPO, polyphenol oxidase; POD, peroxidase. Statistical significance of DEGs was defined using adjusted *p*-value < 0.05 and |fold-change| > 1.5 as a cut-off.

Catechins play an essential role in tea polyphenols and have an important influence on the quality and function of tea [26]. In this research, the content of flavan-3-ol (catechins) gradually decreased with the increase in withering time, due to the downregulation of the DEGs involved in catechins metabolism (Figure 3a). The different DEGs encoding enzymes involved in the biosynthesis and oxidation of catechins were examined, and 18 DEGs were detected, including gene encoding PAL (phenylalanine ammonia-lyase), C4H (cinnamate-4-hydroxylase), TT4 (chalcone synthase), TT5 (chalcone isomerase), TT7 (flavonoid 3’-monooxygenase), F3H (flavanone 3-hydroxylase), DFR (dihydro flavonol 4-reductase), ANR (anthocyanidin reductase), and FLS (flavonol synthase) (Figure 3a). The ANR involved in the formation of flavan-3-ol monomers, such as C, EC, and EGC, gradually decreased (Figure 3a). The reduction in quercetin content was caused by its associated DEGs (CSS0007745, novel.8182), which demonstrated downregulation during withering, especially in CK and W4h (Figure 3a).

The three genes encoding DFR (CSS0000672, CSS0042695, and CSS0003473) were upregulated as withering time increased, leading to the synthesis of dihydrokaempferol, dihydroquercetin, and dihydromyricetin into leucopelagonidin, leucocyanidin, and leucodelphinidin, respectively. DFR has been reported as a core enzyme in the synthesis of nongalloylated catechins, and its catalytic products affect downstream metabolites [26]. Genes encoding ANR (CSS0041663, CSS0033195), involved in the formation of the catechins showed a downregulation trend, and some upstream genes, including TT4, TT5, and F3H, involved in the biosynthesis of catechins, were downregulated as withering time increased (Figure 3a), which could resulted in reduction in catechin content during withering [6]. The enzyme UDP-glucose:galloyl-1-O- β-D-glucosyltransferase (UGGT) converted gallic acid (GA) into β-glucogallin (an active acyl donor), then catalyzed the transfer of β-glucogallin to the third position of the C ring in nongalloylated catechin, in the presence of epicatechin:1- O-galloyl- β-D-glucose O galloyltransferase (ECGT) (Figure 3a) [38]. Additionally, the conversion of catechins into theaflavins in the presence of polyphenol oxidase (PPO) and peroxidase (POD) could reduce the content of catechins [6]. In our research, PPO and POD showed an increasing trend during withering time (Figure 3c). The genes encoding leucocyanidin reductase (LAR), which catalyzes leucocyanidin and leucodelphinidin to produce catechin (C) and galloachachin (GC), respectively, were significantly (*p* < 0.05) downregulated. Thus, the increase in C content was due to the degradation of galloylated catechins/ester–catechins to produce simple catechins [39]. In short, the decrease in catechin content depends on two main sources—one is the oxidation of catechin into theaflavins (TFs) (Figure 3c) and the other is the inhibition of the genes involved in catechin biosynthesis—as also pointed out in previous research [40]. 

#### 3.3.3. Characterization of the DEGs Involved in Amino Acid Metabolism During the Withering of Black Tea

**Figure 4:**
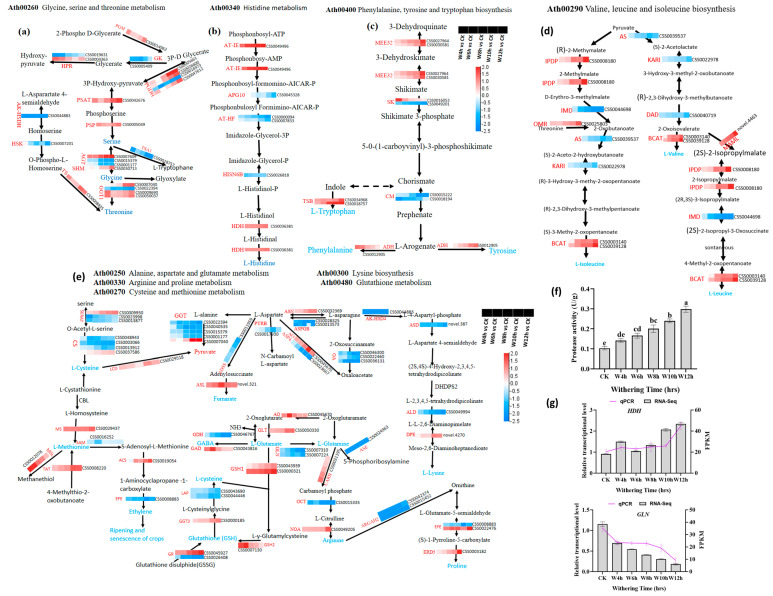
The differentially expressed genes (DEGs) involved in amino acid formation during the withering process. (**a**) Glycine, serine and threonine metabolism; (**b**) histidine metabolism; (**c**) phenylalanine, tyrosine, and tryptophan biosynthesis; (**d**) valine, leucine, and isoleucine biosynthesis; (**e**) alanine, aspartate, and glutamate metabolism; arginine and proline metabolism; cysteine and methionine metabolism; lysine biosynthesis; (**f**) protease activity; (**g**) the validation of RNA-seq by RT-qPCR. The column charts represent values of RNA-seq, and the lines represent RT-qPCR values. Statistical significance of DEGs was defined using adjusted *p*-value < 0.05 and |fold-change “FC” | > 1.5 as a cut-off.

In tea, the free amino acid count in 1–4% of dry weight richly contributes to the taste of tea, especially umami and sweetness in tea infusions [41]. In this study, we identified the differentially expressed amino acid metabolic genes in various pathways. During withering, serine increased as withering time increased due to its synthesis from phosphoserine in the presence of phosphoserine phosphatase (PSP), which was upregulated (Table S1). On the other hand, alanine-glyoxylate transaminase carried out transamination from alanine and glyoxylate to glycine and pyruvate. The serine hydroxymethyltransferase (SHM) increased with withering time from CK (0.004 mg/g DW) to W12h (0.008 mg/g DW). Threonine synthase (TS) and glutamate-glyoxylate aminotransferase 1 (GGT1) were the enzymes responsible for synthesizing threonine, and their expression increased with withering time (Figure 4a and Table S1). Histidinol dehydrogenase (HDH) is well-known to be responsible for the synthesis of histidine at the final step. The expression of HDH was upregulated and increased the histidine content during withering process (Figure 4b and Table S1). Finally, phenylalanine and tyrosine were synthesized from the arogenate compound by arogenate dehydrogenase (ADH); the expression of ADH was upregulated, which could have triggered the increase in phenylalanine and tyrosine observed in this study. Tryptophan content gradually increased with withering, and it was synthesized from indole by the tryptophan synthase beta chain (TSB) (Figure 4c and Table S1). The DEGs (CSS0003140, CSS0039128) encoding branched-chain amino acid aminotransferase (BCAT) contributed to the synthesis of valine, leucine, and isoleucine, and the expression of BCAT was upregulated as withering time increased despite the downregulation of upstream genes. The expression of BCAT increased the relative content of valine, leucine, and isoleucine (Figure 4d and Table S1). This result was consistent with previous studies [25]. The DEGs (CSS0028325, CSS0013573) encoding asparaginase/beta-aspartyl-peptidase (ASPGB), which are responsible for converting asparagine into aspartate, were downregulated, while the gene (CSS0032369) encoding asparagine synthase (ANS) that converted aspartate into asparagine and vice versa was upregulated. As result, asparagine content gradually increased from CK (0.016 mg/g) to W12h (0.036mg/g) (Figure 4e and Table S1). The DEGs (CSS0007310, CSS0007224) encoding glutamine synthetase (GLN), which contribute to the synthesis glutamine, showed downregulation, but the hydrolysis of glutamate can increase the content of glutamine. Arginine was synthesized from citrulline by the DEG (CSS0049205) encoding nitric-oxide synthase (NOA), which was upregulated and triggered the arginine content to increase from 0.021 mg/g to 0.037mg/g (Figure 4e and Table S1). Proline synthesized from (s)-1-pyrroline-5-carboxylate in the presence of proline dehydrogenase (ERD5) showed the same trend as arginine, but proline increased in the following order: CK (0.028 mg/g DW) < W4h (0.041 mg/g DW) < W6h (0.046 mg/g DW) < W8h (0.049 mg/g DW) < W10h (0.054 mg/g DW) < W12h (0.062 mg/g DW) (Figure 4e and Table S1). The increase in proline level may be due to the expression of ERD5, which was upregulated during withering. Theanine, known as the most abundant amino acid in tea, gradually decreased with withering time. Previous research showed that theanine is synthesized in the roots and is transported through the leaves; therefore, plucking leaves from the plant reduced its synthesis [42]. Generally, the free amino acids are formed from their polymers/protein by the intervention of protease via the hydrolysis of peptide. In this study, protease activity increased with withering time (Figure 4f). According to previous research, amino acids were accumulated due to the intervention of protease activities to degrade protein and the synthesis of amino acids during the withering process [39].

#### 3.3.4. Characterization of the DEGs Involved in Starch Metabolism During the Withering of Black Tea

**Figure 5:**
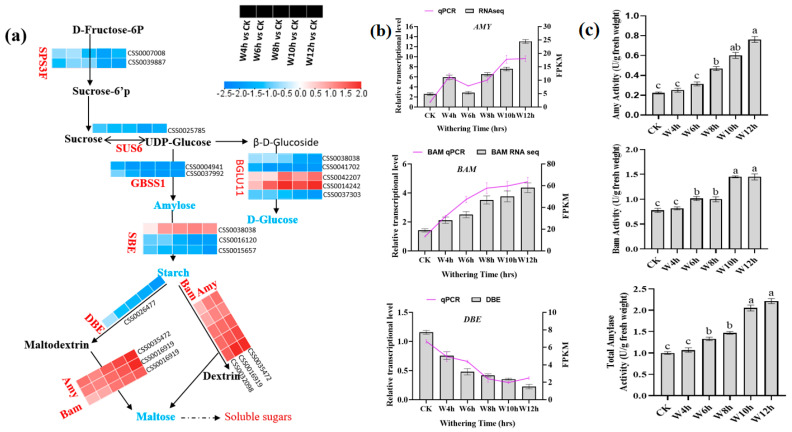
Dynamic changes in metabolites. (**a**) The differentially expressed genes (DEGs) involved in starch and sucrose metabolism during withering process. (**b**) Validation of RNA−seq by RT−qPCR; column charts represent values of RNA−seq, and lines represent RT−qPCR values. (**c**) Amylase activities. SPS3F, sucrose phosphate synthase 3F; SUS6, sucrose synthase 6; BGLU11, beta glucosidase 11; GBSS1, granule-bound starch synthase 1; SBE, starch−branching enzyme; DBE, isoamylase/debranching enzyme; AMY, alpha-amylase; BAM, beta-amylase. Statistical significance of DEGs was defined using the adjusted *p*−value < 0.05 and a |fold-change “FC” | > 1.5 as a cut-off.

Based on KEGG pathways analysis, our study reveals that starch and sucrose metabolism were the first enriched pathways (Figure 2c). At the withering stage, a total of 17 DEGs encoding the essential enzymes related to the metabolism of starch and sucrose were found, such as genes (CSS0035472, CSS0016919) encoding alpha-amylase (AMY), which acted as endohydrolase, and the gene (CSS0032098) encoding beta-amylase (BAM), which acted as exohydrolase; AMY and BAM were upregulated during withering (Figure 5a). Consequently, they influenced the increase in starch monomers, such as maltose, as withering time increased. These genes play a key role in the decomposition of starch during black tea processing, as revealed in our previous studies [6]. AMY triggers the decomposition of starch to produce malto-oligosaccharides, while BAM catalyzes the α-1,4-glucosidic linkages of starch to produce β-maltose derived from the non-reducing part [43]. Other research showed that withering dehydration stress initiates gene expression and causes the starch-related genes to respond to the water deficit by providing energy and carbon to sustain photosynthesis process [11,43].

#### 3.3.5. Characterization of the DEGs Involved in Terpenoid Backbone Biosynthesis During the Withering of Black Tea

**Figure 6:**
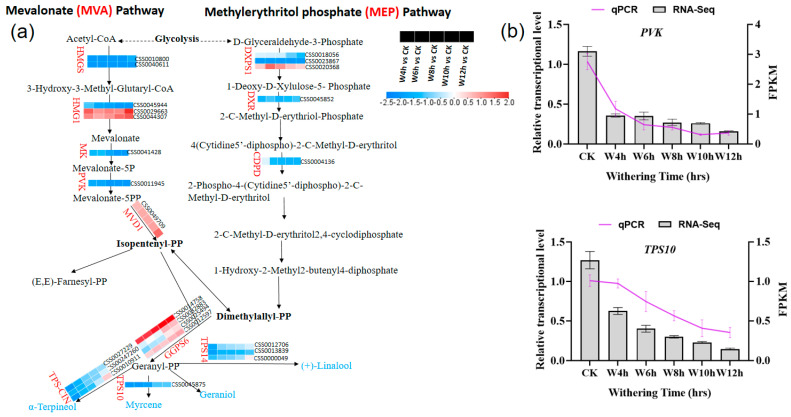
(**a**) The differentially expressed genes (DEGs) involved in terpenoid backbone biosynthesis during withering process; (**b**) Validation of RNA−seq by RT−qPCR; column charts represent values of RNA−seq, and lines represent RT−qPCR values. HMGS, hydroxymethylglutaryl−CoA synthase; HMG1, hydroxy methylglutaryl CoA reductase 1; MK, mevalonate kinase; PVK, phosphomevalonate kinase; MVD1, mevalonate diphosphate decarboxylase 1; DXPS1, 1−deoxy-D-xylulose 5−phosphate synthase 1; DRX, 1−deoxy-D-xylulose 5-phosphate reductoisomerase; CDPD, 4−(cytidine 5’-phospho)-2-C-methyl-D-erithritol kinase; GGPS6, geranylgeranyl pyrophosphate synthase 6; TPS10, terpene synthase 10; TPS14, terpene synthase 14; TPS−CIN, terpene synthase-like sequence-1,8-cineole. Statistical significance of DEGs was defined using the adjusted *p*-value < 0.05 and |fold-change “FC” | > 1.5 as a cut-off.

In this study, we investigated the DEGs involved in the biosynthesis of terpenoids during the black tea withering process. Plant terpenes are synthesized from two isomeric five-carbon precursor and have isopentenyl diphosphate (IPP) as a common precursor (Figure 6a). Terpene biosynthesis is catalyzed by a class of enzymes called terpene synthases (TPSs), and a previous study demonstrated that the withering process upregulates certain TPS-related genes [44]. The terpenoids are synthesized through two different independent pathways, one is the mevalonate (MVA) pathway in cytosol and the second one is the methylerythritol phosphate (MEP) pathway in plastid [45]. After isomerization, IPP produced dimethylallyl pyrophosphate (DMAPP), which can be used as a substrate for the biosynthesis of hemiterpenes or combined with one IPP unit to generate geranyl diphosphate (GPP) and was the precursor of monoterpenes [45]. The MEP and MVA biosynthesis pathways are connected by a metabolic process mediated by unknown transporters. The MEP pathway largely produces monoterpenes, which account for 53% all floral note terpenoids, and diterpenes, which account for 1% all floral note terpenoids, whereas the MVA pathway primarily produces sesquiterpenes, which account for 28% of all floral terpenoids [44].

In the mevalonate (MVA) pathway, different DEGs associated with HMGS (hydroxymethylglutaryl-CoA synthase), HMG1 (hydroxy methylglutaryl CoA reductase 1), MV (mevalonate kinase), and PVK (phosphomevalonate kinase) were identified (Figure 6a). Previous research showed that HMGS is considered a rate-limiting enzyme in the MVA pathway, and it catalyzes the synthesis of mevalonic acid from hydroxy methylglutaryl CoA [46]. In our study, HMG1 coding genes (CSS0029663, CSS0044307) were upregulated, thereby leading to the increased synthesis of terpenoids, as previously shown [47]. On the other hand, the MEP pathway DEGs corresponding to DXPS1 (1-deoxy-D-xylulose 5-phosphate synthase 1), DXR (1-deoxy-D-xylulose 5-phosphate reductoisomerase), and CDPD (4-(cytidine 5’-phospho)-2-C-methyl-D-erithritol kinase) were identified at the upstream of isopentenyl-PP (Figure 6a). Gene encoding MVD1 was upregulated in order to produce IPP that can further interchangeably convert into DMAPP (Figure 6a). The GGPS6 (geranylgeranyl pyrophosphate synthase 6) encoded by gene (CSS0012597) was upregulated as withering time increased to form Geranyl-PP (precursor for monoterpenes), while others were downregulated. Terpene synthase 10, 14, and TPS-CIN (terpene synthase-like sequence-1,8-cineole) were downregulated in withered samples, but their expression increased over time and are responsible for forming myrcene, linalool, and α-terpineol, respectively; these compounds are floral scents [48]. It was suggested that some flavor compounds can be formed from enzymatic and other chemical reactions.

#### 3.3.6. Characterization of the DEGs Involved in α-Linolenic Acid Metabolism During the Withering of Black Tea

**Figure 7:**
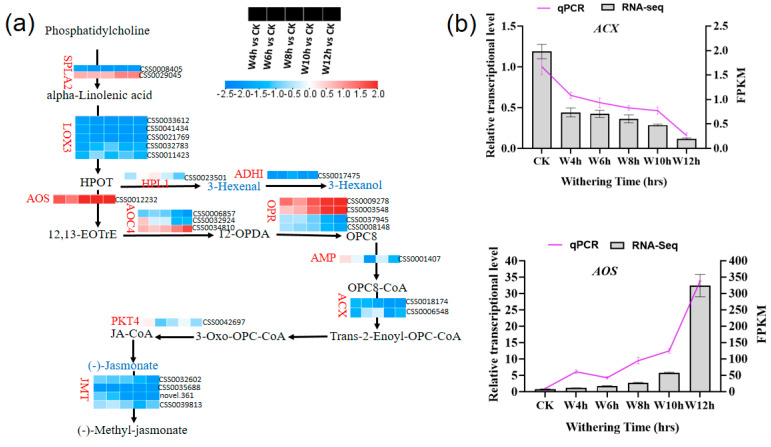
(**a**) Simplified model of DEGs involved in α−linolenic acid metabolism during withering process. (**b**) Validation of RNA-seq by RT−qPCR; column charts represent values of RNA−seq, and lines represent RT−qPCR values. PLA2−ALPHA, secretory phospholipase A2; LOX3, lipoxygenase; HPL1, hydroperoxide lyase 1; ADH1, alcohol dehydrogenase class−P, AOS, hydroperoxide dehydratase; AOC4, allene oxide cyclase 4; OPR, Oxophytodienoic acid reductase; AMP, AMP-dependent synthetase and ligase family protein; ACX, acyl−CoA oxidase; PKT4, peroxisomal 3−ketoacyl−CoA thiolase 4; JMT, jasmonate O−methyltransferase. Statistical significance of DEGs was defined using the adjusted *p*−value < 0.05 and |fold-change “FC” | > 1.5 as a cut−off.

Fatty acids are vital precursors of volatile organic compounds in tea, especially green leaf volatiles [49]. The α- linolenic acid (ALA), an unsaturated fatty acid, was formed from phosphatidylcholine in the presence of gene encoding SPLA2 (secretory phospholipase A2). Lipids under oxygenation reaction with LOX3 (lipoxygenase) formed lipid hydroperoxide in the presence of HPL1(hydroperoxide lyase 1), then lipid hydrogen peroxides were subsequently reduced to their corresponding alcohols (leaf alcohol) by ADHI (alcohol dehydrogenase) (Figure 7a). Based on the transcriptome data, the DEGs associated with LOX3, HPL1, and ADHI were downregulated during the withering process which resulted in a decrease of leaf alcohols content like hexanol (Figure 7a).

Leaf alcohol has a greenish and grassy aroma, which is undesirable in black tea flavor characteristics [35]. The oxidation of lipids into volatile compounds was linked to two common pathways, namely oxidation reaction triggered by free radicals and lipid oxidation catalyzed by lipoxygenase [35]. The DEGs encoding jasmonate O-methyltransferase (JMT), which contributed to the synthesis of (-)-Methyl-jasmonate (floral-fruity like aroma), were downregulated but not remarkably changed (Figure 7a).

According to the findings, the transcripts assembled were verifiable, and the primer pairs used were appropriate for use in the expression tests. There was correlation in expression patterns between the RNA-Seq data and the RT-qPCR results for chosen genes, indicating that the unigenes derived from the assembled transcriptome were reliable.

**Figure 8:**
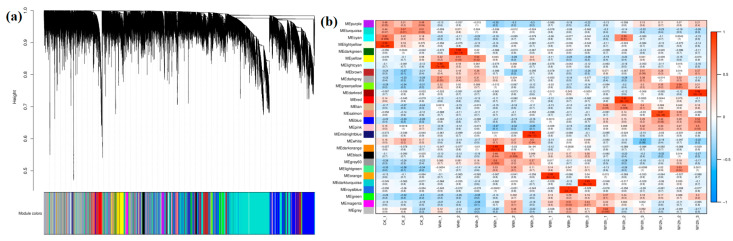
Weighted gene co-expression network analysis (WGCNA) of the genes during the withering process. (**a**) Cluster dendrogram displaying the hierarchical cluster tree with each leaf representing a single gene and the co−expression modules revealed by WGCNA. (**b**) The correlations between samples and modules where every row represents a single module. The color and number of each cell at the row−column intersection indicate the correlation coefficient of the module and sample.

To further understand whether the gene modules identified as shown in Figure 8 were related to aforementioned tea metabolites biosynthesis pathways, we analyzed the DEGs and co-expression networks using WGCNA. The 28 gene co-expression modules were found and are shown in Figure 8a (cluster dendrogram labeled with different colors). The modules were relatively correlated with the metabolites associated with the synthesis of catechins, α-linolenic acid metabolism, amino acid metabolism, and starch metabolism throughout the withering process. Interestingly, the ME turquoise co-expression module contained 8329 genes that gradually decreased in their expression as withering time increased, including genes associated with the synthesis of the catechins such as CSS0041448 encoding PAL, CSS0002737 encoding C4H, CSS0030597 encoding TT4, CSS0050436 encoding TT5, CSS0016177 and CSS0019002 encoding F3H, and CSS0033195 encoding ANR. ME turquoise also showed the genes involved in the α-linolenic acid metabolism, including CSS0033612, CSS0011423, CSS0032783, and CSS0021769 encoding LOX 3; CSS0017475 encoding ADHI; CSS0008148 and CSS0037945 encoding OPR; CSS0006548 encoding ACX; and CSS0032602, CSS0035688, and CSS0039813 encoding JMT. However, some other modules showed the trend of upregulated genes during the withering process. For example, the ME blue module contained 5529 genes, including genes associated with the amino acid metabolism, such as CSS0012905 encoding ADH, CSS0008180 encoding IPDP, CSS0009950 and CSS0013877 encoding SERAT, CSS0012078 encoding MGL, CSS0008220 encoding TAT, CSS0050330 encoding GLT, CSS0043816 encoding GAD, CSS0000185 encoding GGT3, CSS0045927 encoding GR, and CSS0003182 encoding ERD5. The ME blue module also showed CSS0035472 and CSS0016919 encoding AMY, which was identified from the starch metabolism, while the ME green module contained 1030 genes, including CSS0032098 encoding BAM, which was identified from the starch metabolism. Therefore, based on gene expression profiling, WGCNA has proven to be the most powerful approach for studying gene correlations, finding modules among highly correlated genes, and connecting modules to phenotypic features [50]. One module can be associated with more components, and one component can be regulated by various modules [50]. This result demonstrated that the genes with the same expression trend were identified in the same module and were relatively well correlated with the metabolites.

## 4. Conclusions

Our study revealed that the numbers of DEGs for the comparative combination of withering time increased from 3634 (W4h) to 5736 (W12h), compared with CK (0h). Some DEGs play direct or indirect roles in the formation of black tea quality metabolites. The reduction in catechins could be the result of the downregulation of the DEGs involved in catechin metabolism. An increase in protease activities and the upregulation of DEGs involved in amino acid metabolism triggered the increase in amino acids in tea; the same trend was observed in the metabolism of starch in the presence of AMY and BAM. RNA-Seq analysis showed that terpenes biosynthesis during the withering process enhanced the aroma compounds of black tea via the synthesis of floral and fruit note metabolites, while the reduction in the greenish/grassy aroma was driven by α-linolenic acid metabolism. The weighted gene co-expression network analysis (WGCNA) of DEGs showed that one module can be linked with multiple traits and vise vasa. Studying the genes associated with tea metabolites and enzyme activities during the withering process provides valuable knowledge that could improve the withering process for the quality control of the product’s standard. This research provides new insights into the formation of black tea quality attributes at the withering level.

## Data Availability

The original contributions presented in the study are included in the article/supplementary material, further inquiries can be directed to the corresponding authors.

## Supplementary Materials

The following supporting information can be downloaded at: https://www.mdpi.com/article/10.3390/foods13233977/s1. Table S1: Differential non-volatile metabolites during withering (mg/g DW); Table S2: Dynamic changes in the volatile compound during withering (ng/g-dw); Table S3: Primer sequence used for qRT-PCR; **Table S4**: Data quality for parametric transcriptome sequencing; Figure S1: Changes in total volatile compounds during withering process of black tea (ng/g-dw).
