# Supplementary material for: Integrated Transcriptomic and Metabolomic Analyses Reveal Changes in Aroma- and Taste-Related Substances During the Withering Process of Black Tea
Source: Foods. 2024 Dec 9;13(23):3977. doi: 10.3390/foods13233977 (PMC11641482; doi:10.3390/foods13233977)
Supplement: Supplementary file 1 [file foods-13-03977-s001.zip › foods-3275585-supplementary.pdf]

Supplemental Data

Table S1 Differential non-volatile metabolites during withering

S/N	Compounds	RT (Min)	Relative content (mg/g DW)						MS2 Fragments	Ion Monitored
	**Catechins/Flavan 3-ols**		**CK**	**W4h**	**W6h**	**W8h**	**W10h**	**W12h**		
1	(-)-gallocatechin/GC	5.34	0.291±0.009a	0.279±0ab	0.268±0.013ab	0.271±0.005ab	0.245±0.019b	0.206±0.012c	223,195,163,139,95	[M+H]+
2	(-)-Epigallocatechin/EGC	6.94	1.694±0.140a	1.678±0.052a	1.631±0.071ab	1.598±0.025ab	1.422±0.174b	1.255±0.040c	223,195,163,139,95	[M+H]+
3	Theasinensin A	7.01	0.038±0.003c	0.049±0.001ab	0.058±0.001b	0.070±0.002a	0.059±0.002b	0.052±0.002ab	271,285,255,229,157	[M+H]+
4	(+)-Catechin/C	7.2	0.176±0.007c	0.199±0.007bc	0.215±0.011bc	0.237±0.005b	0.235±0.012b	0.263±0.006a	207,139,123,95,55	[M+H]+
5	Epigallocatechin-(4beta->8)-epicatechin-3-O-gallate ester	7.63	0.007±0b	0.009±0a	0.008±0ab	0.008±0ab	0.007±0b	0.007±0b	443,425,305,151,123	
6	Theasinensin F	7.91	0.029±0.002c	0.032±0.001c	0.034±0.004c	0.049±0.002ab	0.048±0.002ab	0.039±0.001b	271,285,255,229	[M+H]+
7	Epigallocatechin gallate/EGCG	8.55	2.309±0.155a	2.010±0.172b	1.855±0.171ab	1.705±0.077c	1.453±0.124c	1.405±0.081c	289,205,153,123,65	[M+H]+
8	Epicatechin /EC	9.11	1.167±0.055c	1.373±0.036ab	1.405±0.085a	1.266±0.017bc	1.168±0.078c	1.147±0.088c	207,139,123,95,55	[M+H]+
9	(-)-Gallocatechin gallate/GCG	9.36	0.264±0.024a	0.259±0.003ab	0.249±0.003ab	0.245±0.013ab	0.235±0.015b	0.206±0.016c	289,153,139,123,65	[M+H]+
10	(-)-Epicatechin gallate/ECG	11.2	0.949±0.043a	0.905±0.031ab	0.844±0.034bc	0.859±0.027bc	0.804±0.045c	0.790±0.043c	291,273,207,153,77	[M+H]+
11	Catechin gallate/CG	11.82	0.920±0.093a	0.905±0.031ab	0.844±0.035abc	0.846±0.015abc	0.811±0.058bc	0.791±0.050c	291,273,207,153,77	[M+H]+
	**Amino acids**									
12	L-Glutamine	0.83	0.015±0d	0.016±0.001c	0.017±0c	0.017±0c	0.020±0.002b	0.030±0.004a	130,84,56	[M+H]+
13	L-Lysine	0.83	0.013±0.001d	0.015±0d	0.017±0.001c	0.017±0c	0.019±0.001b	0.026±0.001a	102,84,56	[M+H]+
14	L-Histidine	0.83	0.003±0b	0.004±0b	0.004±0b	0.005±0ab	0.005±0ab	0.006±0a	110,93,83,56	[M+H]+
15	L-Arginine	0.96	0.021±0c	0.025±0.001bc	0.027±0.002bc	0.030±0b	0.030±0.001b	0.037±0.001a	158,130,116,70,60	[M+H]+
16	L-Asparagine	0.99	0.016±0d	0.021±0.002cd	0.025±0c	0.028±0.001bc	0.031±0.002b	0.036±0.003ab	87,74,70,60	[M+H]+
17	L-Serine	1.02	0.004±0b	0.005±0b	0.005±0b	0.006±0a	0.007±0.001a	0.008±0a	158,105,89,73,44	[M+H]+
18	L-Glutamic acid	1.05	0.038±0.003a	0.030±0b	0.024±0bc	0.024±0bc	0.023±0.001c	0.022±0.001c	130,102,84,56	[M+H]+
19	L-Proline	1.08	0.028±0.001e	0.041±0d	0.046±0.001c	0.049±0.001c	0.054±0.002b	0.062±0.003a	116,72,70,44	[M+H]+
20	L-Valine	1.11	0.003±0b	0.004±0b	0.004±0b	0.005±0b	0.006±0a	0.007±0a	94,72,53,55	[M+H]+
21	L-Theanine	1.23	0.129±0.016a	0.119±0.005ab	0.102±0.005b	0.091±0.004bc	0.081±0.005c	0.079±0.004c	158,129,84,56	[M+H]+
22	L-Tyrosine	1.85	0.012±0c	0.014±0c	0.015±0bc	0.016±0bc	0.018±0.001b	0.024±0.001a	136,123,119,95,91,27	[M+H]+
23	L-Isoleucine	2.18	0.007±0d	0.010±0c	0.011±0.001c	0.013±0.001b	0.013±0b	0.015±0.001a	86,69,57	[M+H]+
24	L-Leucine	2.28	0.008±0d	0.010±0.001d	0.012±0.001c	0.013±0.001c	0.016±0.001b	0.019±0.001a	86,69,55	[M+H]+
25	L-Phenylalanine	4.7	0.087±0.005e	0.146±0.002d	0.159±0.005cd	0.168±0.006c	0.217±0.011b	0.274±0.011a	120,103,77,51	[M+H]+
26	L-Tryptophan	6.3	0.004±0b	0.004±0b	0.005±0ab	0.006±0ab	0.006±0a	0.007±0a	188,170,146,118	[M+H]+
	**Flavonoid/Flavonoid glycosides**									

27	Procyanidin B1	6.63	0.049±0.002d	0.053±0.002d	0.060±0.003c	0.069±0.001b	0.070±0.005b	0.069±0.003a	374,289,260,127,90	[M+H]+
28	(2R,3S,4S)-leucocyanidin	6.74	0.080±0.005a	0.079±0.001a	0.074±0.002a	0.077±0a	0.068±0.004ab	0.059±0b	305,287,255,245,143,119	[M-H]-
29	Dihydromyricetin	8.28	0.001±0b	0.002±0ab	0.002±0ab	0.003±0a	0.003±0a	0.003±0a	320,302,151,135	[M+H]+
30	Benzyl β-glucoside	8.63	0.006±0a	0.006±0a	0.005±0b	0.005±0b	0.004±0b	0.003±0b	265,234,169,129,117	[M+H]+
31	Dihydroquercetin	9.38	0.010±0b	0.012±0ab	0.015±0a	0.014±0.001a	0.012±0ab	0.012±0ab	305,287,139,125,99	[M+H]+
32	2-Phenylethyl primeveroside	9.77	0.021±0.001a	0.018±0b	0.017±0b	0.017±0b	0.015±0.001c	0.015±0c	401,275,123	[M+H]+
33	(-)-Epiafzelechin	10.77	0.153±0.002b	0.171±0.009a	0.141±0.007bc	0.131±0.003cd	0.125±0.011d	0.121±0.005d	257,191,163,139,107,95	[M+H]+
34	Quercetin 3-sophorotrioside	11.47	0.011±0a	0.010±0a	0.009±0ab	0.009±0ab	0.008±0b	0.008±0b	319,145,127,85,71	[M+H]+
35	Myricetin 3-O-galactoside	11.95	0.054±0.002a	0.048±0.004b	0.046±0.001b	0.045±0b	0.040±0.001c	0.037±0.005c	319,127,85,61	[M+H]+
36	Quercetin-7-O-β-D-glucopyranoside	12.88	0.039±0.001a	0.034±0.002b	0.027±0.002c	0.027±0.002c	0.024±0.001cd	0.021±0.001d	465,303, 257,153,121	[M+H]+
37	Quercetin 3-Rutinoside-7-Glucoside	13.16	0.108±0.010b	0.123±0.006a	0.112±0.003ab	0.111±0.007ab	0.103±0.007b	0.096±0.011b	303,145,129,97,71	[M+H]+
38	Isovitexin-2''-glucoside	13.25	0.004±0a	0.004±0a	0.003±0ab	0.003±0ab	0.003±0ab	0.002±0c	433,415,367,337,313	[M+H]+
39	Hyperoside	13.81	0.046±0.001a	0.045±0.002a	0.043±0.001a	0.042±0.002ab	0.036±0.002b	0.035±0.003b	335,298,232,153,81	[M+H]+
40	Quercetin 3-rutinoside	14.13	0.063±0.008a	0.057±0.002b	0.057±0.006b	0.053±0.003bc	0.049±0.003c	0.044±0.003c	449,329,287,85	[M+H]+
41	Myricetin	15.02	0.006±0.001a	0.005±0b	0.005±0b	0.005±0b	0.005±0b	0.004±0c	273,217,165,153,137	[M+H]+
42	Quercetin-3-O-D-glucosyl]-(1-2)-L-rhamnoside	15.08	0.018±0.001a	0.017±0.001a	0.016±0ab	0.016±0ab	0.015±0b	0.015±0.001b	449,413,369,303,85	[M+H]+
43	Astragaline	15.14	0.054±0.003bc	0.056±0.001b	0.062±0.003a	0.054±0.001bc	0.050±0.003c	0.049±0.006c	288,214,153,127,85	[M+H]+
44	Kaempferol 3-O-rhamninoside	15.25	0.008±0a	0.008±0a	0.007±0b	0.007±0b	0.007±0b	0.007±0b	287,153	[M-H]-
45	Kaempferol 3-rutinosidea	15.41	0.102±0.008a	0.095±0.010ab	0.090±0.011b	0.091±0.002b	0.082±0.005bc	0.074±0.006c	593,431,285, 225,151	[M-H]-
46	Quercitrin	16.14	0.004±0a	0.003±0ab	0.003±0ab	0.003±0ab	0.002±0b	0.002±0b	303,129,85,71,57	[M+H]+
47	Kaempferol-3-O-(6'''-trans-P-coumaroyl-2''-glucosyl) rhamnoside Flavono	16.49	0.009±0a	0.008±0ab	0.008±0ab	0.008±0ab	0.007±0b	0.007±0b	449,287,285	
48	Quercetin	15.96	0.004±0a	0.003±0ab	0.003±0ab	0.002±0b	0.002±0b	0.002±0b	229,153,69	[M+H]+
	**Theaflavins**									
49	Theaflavin-3-gallate	15.87	0.014±0.001c	0.017±0.001b	0.018±0.002b	0.020±0ab	0.022±0.001ab	0.026±0.002a	379,277,139	[M+H]+
50	Theaflavine-3,3'-digallate	15.93	0.018±0.001d	0.021±0.002cd	0.025±0.003c	0.031±0.003ab	0.034±0.002ab	0.037±0.001a	699,529,333,277	[M+H]+
51	Theaflavin-3'-gallate	15.96	0.013±0.001c	0.021±0.002b	0.026±0.002a	0.023±0.001ab	0.019±0.002b	0.018±0b	579,333,277,139	[M+H]+
52	Theaflavin	15.9	0.009±0.001d	0.012±0.001c	0.013±0b	0.019±0.001a	0.014±0.001b	0.012±0c	427,277,139	[M+H]+
	**Alkaloids**									

53	Betaine	1.07	0.009±0a	0.009±0a	0.007±0ab	0.007±0ab	0.006±0b	0.006±0b	135,118,99,87,58	[M+H]+
54	3-Methylxanthine	4.82	0.022±0.001a	0.019±0b	0.015±0.001c	0.014±0.001cd	0.013±0.001cd	0.010±0.001d	167,150,123,110,95	[M+H]+
55	Caffeine	8.42	6.855±0.400a	6.450±0.244ab	5.655±0.228bc	5.484±0.175bc	4.982±0.390cd	4.126±0.211d	138,110,83,56	[M+H]+
56	Theobromine	6.02	0.133±0.010a	0.106±0.003b	0.091±0.004c	0.074±0.001d	0.066±0.007d	0.069±0.004d	163,138,122,83,67,56	[M+H]+
57	Indole	6.22	0.003±0.002b	0.004±0.001b	0.005±0b	0.006±0ab	0.006±0ab	0.008±0.001a	118,91,72,59	[M+H]+
	**Organic&Phenolic acids**									
58	Shikimic acid	0.93	0.013±0.001c	0.018±0a	0.015±0b	0.014±0.001bc	0.013±0c	0.013±0c	158,84,56	[M+H]+
59	Salicylic acid	1.09	0.007±0.001c	0.009±0bc	0.009±0bc	0.011±0b	0.012±0.001b	0.017±0a	139,121,95,83,69	[M+H]+
60	D-(-)-Quinic acid	1.13	0.004±0c	0.005±0c	0.005±0c	0.006±0b	0.006±0b	0.008±0a	147,129,111,77,65,55	[M+H]+
61	Gallic acid	2.49	0.006±0c	0.007±0c	0.008±0b	0.009±0b	0.010±0.001ab	0.013±0.001a	153,125,109,81,53	[M+H]+
62	Chlorogenic acid	7.98	0.004±0c	0.005±0c	0.006±0c	0.006±0b	0.007±0b	0.009±0a	163,145,135,117,89	[M+H]+
63	Ferulic acid	8.21	1.043±0.006a	1.006±0.026ab	0.939±0.023b	0.953±0.014b	0.860±0.060c	0.793±0.053d	195,138,110,69	[M+H]+
64	p-Coumaric acid	10.68	0.005±0c	0.006±0c	0.007±0c	0.007±0b	0.008±0b	0.009±0a	147,119,91,65	[M+H]+
65	Caffeic acid	19.19	0.005±0b	0.006±0b	0.006±0b	0.007±0a	0.007±0a	0.008±0a	163,135,107,89,79	[M+H]+
	**Others**									
66	Theogallin	3.84	0.116±0.005a	0.100±0.009b	0.098±0.010b	0.092±0.013bc	0.088±0.007bc	0.082±0.007c	343,191,169,125	[M-H]-
67	4-Indolecarbaldehyde	6.22	0.004±0c	0.006±0c	0.007±0b	0.009±0b	0.009±0b	0.011±0a	146,118,69	[M+H]+
68	4-Hydroxybenzaldehyde	10.93	0.007±0a	0.006±0a	0.007±0a	0.006±0a	0.005±0b	0.006±0b	123,105,95,67,51	[M+H]+

Note: W0h /CK, W4h, W6h, W8h, W10h, and W12h stand for the withering time set at 0h, 4hrs, 8hrs, 8hrs, 10hrs, and 12hrs, respectively. RT, retention time. Results are presented as mean ±SD (n=3), and a significant difference is shown by different letters in the same column with a threshold p<0.05 according to Duncan's test.

Table S2 Dynamic changes in the volatile compound during withering

S/N						ng/g-dw					
	**Compounds**	**Molecular Formula**	**RT**	**RI-LIB**	**RI-SAMPLE**	**CK**	**W4h**	**W6h**	**W8h**	**W10h**	**W12h**
	**Alcohols/Terpene Alcohols**										
**1**	(Z)-Hex-4-en-1-ol	C6H12O	4.55	879	878.77	6.94±0.24a	5.36±0.31b	3.38±0.30c	2.41±0.07d	1.76±0.19e	1.66±0.09e
**2**	trans-2,4-Hexadien-1-ol	C6H10O	5.30	916	920.21	0.06±0.00a	0.05±0.00b	0.04±0.00c	0.03±0.00d	0.03±0.00d	0.03±0.00d
**3**	2-ethyl-2-hexen-1-ol	C8H16O	7.31	996	1007.35	13.2±0.38a	11.66±0.63b	9.82±0.53c	9.48±0.21c	9.28±0.24c	7.79±0.42d

**4**	(E)-6-Methylhept-4-en-1-ol	$C_8H_{16}O$	7.68	1020	1020.43	1.66±0.06a	1.27±0.13b	0.85±0.01c	0.80±0.07c	0.52±0.05d	0.31±0.01e
**5**	(Z)-linalool oxide (furanoid)	$C_{10}H_{18}O_2$	10.12	1086	1093.57	25.70±1.59f	30.06±0.76e	33.81±1.62d	36.94±1.50c	40.81±1.56b	46.11±0.84a
**6**	(E)-linalool oxide (furanoid)	$C_{13}H_{22}O_4$	10.78	1090	1110.21	28.06±1.74d	30.92±0.14cd	32.84±2.39cd	35.91±0.92c	45.15±2.29b	50.72±4.28a
**7**	Linalool	$C_{10}H_{18}O$	11.63	1099	1130.20	110.19±5.29e	134.33±4.71d	143.71±3.89d	163.41±6.61c	185.23±2.39b	196.63±2.53a
**8**	4-Isopropenyl-1-methylcyclohexanol	$C_{10}H_{18}O$	12.05	1153	1139.54	0.02±0.00c	0.03±0.00bc	0.03±0.00bc	0.03±0.00bc	0.06±0.00a	0.04±0.01b
**9**	Phenylethyl alcohol	$C_8H_{10}O$	12.56	1116	1150.46	14.77±0.94d	18.59±1.05c	21.18±2.11bc	23.07±1.55b	24.26±1.02b	31.67±0.92a
**10**	Cis-3-nonen-1-ol	$C_9H_{18}O$	13.81	1156	1175.44	ND	0.03±0.00c	0.05±0.00b	0.05±0.01b	0.08±0.01a	0.07±0.01a
**11**	isocarveol	$C_{10}H_{16}O$	14.13	1186	1181.47	0.05±0.00c	0.06±0.00c	0.08±0.00b	0.13±0.01a	0.10±0.01ab	0.09±0.00b
**12**	(±)Myrtenol	$C_{10}H_{16}O$	14.44	1195	1187.19	0.08±0.00a	0.06±0.01b	0.05±0.00b	0.04±0.00bc	0.03±0.00c	0.03±0.00c
**13**	2,2,6-Trimethyl-6-vinyltetrahydro-2H-furan-3-ol	$C_{10}H_{18}O_2$	14.96	1178	1196.50	0.29±0.02d	0.30±0.05d	0.45±0.03c	0.68±0.01b	0.84±0.12a	0.85±0.11a
**14**	(±)-dihydrocarveol	$C_{10}H_{18}O$	15.35	1192	1203.91	13.52±0.66a	8.46±0.54b	7.79±0.20b	6.43±0.13c	5.84±0.61c	4.60±0.42d
**15**	(Z)-3-decen-1-ol	$C_{10}H_{20}O$	16.51	1225	1226.81	ND	0.56±0.03d	0.65±0.04d	0.83±0.07c	1.07±0.05b	1.36±0.00a
**16**	(3Z)-3,7-Dimethyl-3,6-octadien-1-ol	$C_{10}H_{18}O$	17.23	1240	1240.22	0.01±0.00b	0.01±0.00b	0.02±0.00b	0.03±0.00ab	0.04±0.00a	0.03±0.00ab
**17**	(3Z)-3,7-Dimethyl-3,6-octadien-1-ol/ Isogeraniol	$C_{10}H_{18}O$	17.57	1240	1246.36	0.31±0.02e	0.39±0.06de	0.44±0.01cd	0.53±0.04bc	0.60±0.05b	0.91±0.04a
**18**	Geraniol	$C_{10}H_{18}O$	18.05	1255	1254.83	0.65±0.03d	0.80±0.13d	1.12±0.03c	1.20±0.08c	1.45±0.09b	1.75±0.08a
**19**	2-Butyl-1-octanol	$C_{12}H_{26}O$	19.08	1277	1272.27	0.02±0.00b	0.02±0.00b	0.03±0.00ab	0.04±0.00ab	0.05±0.00a	0.06±0.00a
**20**	2,6-dimethylocta-1,7-diene-3,6-diol	$C_{10}H_{18}O_2$	20.85	1273	1300.00	0.05±0.00c	0.06±0.00c	0.10±0.03b	0.10±0.00ab	0.12±0.02ab	0.13±0.01a

**21**	8-hydroxylinalool	$C_{10}H_{18}O_2$	24.50	1361	1372.14	0.02±0.00b	0.02±0.00b	0.03±0.01ab	0.03±0.00ab	0.03±0.00ab	0.04±0.00a
**22**	2-Hexyl-1-decanol	$C_{16}H_{34}O$	31.38	1504	1501.13	0.14±0.01a	0.10±0.01b	0.07±0.00c	0.07±0.00c	0.07±0.01c	0.06±0.00c
**23**	3,7,11-trimethyldodeca-6,10-dien-1-yn-3-ol	$C_{15}H_{24}O$	34.87	1562	1576.39	0.08±0.01a	0.05±0.00b	0.03±0.00c	ND	ND	ND
**24**	α-cadinol	$C_{15}H_{26}O$	38.40	1653	1668.69	0.67±0.05a	0.56±0.03b	0.51±0.01bc	0.45±0.06c	0.26±0.03d	0.22±002d
**25**	2-Hexadecanol	$C_{16}H_{34}O$	39.37	1702	1723.61	0.11±0.00c	0.12±0.01c	0.17±0.01b	0.18±0.03b	0.29±0.02a	0.19±0.04b
**26**	isocalamendiol	$C_{15}H_{26}O_2$	39.98	1761	1757.46	0.02±0.00b	0.03±0.00b	0.03±0.00b	0.03±0.00b	0.07±0.00a	0.06±0.01a
	**Aldehydes**										
**27**	2-Hexenal	$C_6H_{10}O$	4.4	854	869.66	0.14±0.03a	0.07±0.01ab	0.08±0.01bc	0.04±0.00c	0.04±0.00c	0.03±0.00c
**28**	Heptanal	$C_7H_{14}O$	5.19	901	914.51	0.37±0.01a	0.23±0.03b	0.15±0.02c	0.13±0.00cd	0.13±0.01cd	0.10±0.01d
**29**	Hexanal, 2-ethyl-	$C_8H_{16}O$	5.95	955	951.63	ND	1.41±0.05d	1.81±0.15c	1.75±0.10cd	2.18±0.18b	2.81±0.26a
**30**	(2E)-2-Octenal	$C_8H_{14}O$	8.75	1060	1054.99	1.19±0.07d	1.24±0.05d	1.62±0.26c	2.44±0.19b	2.67±0.10b	3.33±0.21a
**31**	cis-4-decenal	$C_{10}H_{18}O$	15.03	1193	1197.73	1.47±0.05a	0.83±0.13b	0.67±0.00c	0.58±0.03c	0.42±0.04d	0.34±0.03d
**32**	β-Cyclocitral	$C_{10}H_{16}O$	16.90	1220	1234.14	0.01±0.00b	0.01±0.00b	0.01±0.00b	0.02±0.00ab	0.03±0.00a	0.03±0.00a
**33**	10-Undecenal	$C_{11}H_{20}O$	20.72	1288	1298.19	0.10±0.00d	0.11±0.00cd	0.12±0.00c	0.12±0.01c	0.15±0.01b	0.18±0.01a
**34**	2-ethylidene-6-methylhepta-3,5-dienal	$C_{10}H_{14}O$	24.09	1395	1364.61	0.17±0.00a	0.15±0.01b	0.12±0.01c	0.07±0.00d	0.06±0.00d	0.06±0.01d
**35**	E,E-10,12-Hexadecadienal	$C_{16}H_{28}O$	38.9	1702	1698.34	0.12±0.02d	0.13±0.02d	0.19±0.02cd	0.27±0.05c	0.42±0.11b	0.55±0.10a
**36**	(Z)-7-hexadecenal	$C_{16}H_{30}O$	40.3	1798	1766.63	0.01±0.00b	0.02±0.00b	0.02±0.00b	0.03±0.00b	0.06±0.00a	0.04±0.00ab
**37**	2,6-Nonadienal, (E,Z)-	$C_9H_{14}O$	13.55	1155	1170.43	1.04±0.31a	0.79±0.05ab	0.70±0.07bc	0.55±0.05bc	0.45±0.02bc	0.39±0.02c
	**Ketone**										

**38**	6,10-Dimethyl-5,9-undecadien-2-one	C13H22O	29.49	1456	1467.23	0.21±0.01c	0.28±0.01bc	0.38±0.03b	0.55±0.06a	0.49±0.04a	0.35±0.05b
**39**	Geranylacetone	C13H22O	29.61	1453	1469.27	0.05±0.00b	0.05±0.00b	0.06±0.00b	0.08±0.01ab	0.08±0.00ab	0.10±0.00a
**40**	β-Ionone	C13H20O	31.08	1491	1495.97	0.09±0.00d	0.10±0.02d	0.14±0.01cd	0.17±0.01c	0.26±0.04b	0.33±0.05a
**41**	(3E,5Z)-6,10-dimethyl-3,5,9-undecatrien-2-one	C13H20O	33.27	1535	1543.07	0.33±0.02a	0.28±0.02b	0.21±0.03c	0.20±0.03c	0.13±0.02d	0.11±0.02d
**42**	β-Irone	C14H22O	33.70	1551	1552.18	0.02±0.00b	0.02±0.00b	0.03±0.00b	0.04±0.00ab	0.06±0.00ab	0.03±0.00b
**43**	1-(3,5-di-tert-butyl-4-hydroxy-phenyl)-propan-1-one	C17H26O2	37.52	1640	1651.66	ND	ND	ND	0.23±0.00c	0.48±0.02b	0.58±0.02a
**44**	Muscone	C16H30O	41.02	1831	1831.57	0.03±0.01b	0.03±0.00b	0.03±0.00b	0.05±0.00a	0.05±0.00a	0.04±0.00ab
**45**	Oxacycloheptadec-8-en-2-one	C16H28O2	42.38	1925	1917.85	0.16±0.01c	0.21±0.03c	0.24±0.02bc	0.25±0.06bc	0.30±0.03b	0.37±0.03a
**46**	(Z)-Oxacyclononadec-10-en-2-one	C18H32O2	45.35	2183	2168.28	1.23±0.10a	0.99±0.05b	0.52±0.01c	0.37±0.03d	0.27±0.00de	0.20±0.02e
	**Hydrocarbons**										
**47**	trans-7-Methyl-3-octene	C9H18	4.86	896	896.67	ND	ND	1.35±0.27c	1.70±0.13c	2.39±0.27b	3.56±0.12a
**48**	1,5-dimethyl-1,5-cyclooctadiene	C10H16	8.50	1047	1047.31	ND	0.48±0.03d	0.98±0.09c	1.05±0.11c	2.24±0.17b	3.03±0.33a
**49**	(Z)-3,7-dimethylocta-1,3,6,-triene	C10H16	8.60	1038	1050.41	ND	ND	1.37±0.00c	2.33±0.15b	2.65±0.34b	3.58±0.24a
**50**	1,11-Dodecadiene	C12H22	14.29	1179	1184.44	0.13±0.01d	0.16±0.00d	0.17±0.01d	0.31±0.01c	0.52±0.03b	0.63±0.06a
**51**	(Z)-tridec-4-ene	C13H26	20.23	1279	1290.66	0.14±0.00e	0.18±0.01d	0.21±0.00d	0.32±0.01c	0.47±0.02b	0.60±0.01a
**52**	1,2-Epoxydodecane	C12H24O	21.70	1307	1318.85	0.02±0.00b	0.02±0.00b	0.03±0.00ab	0.03±0.00ab	0.05±0.00a	0.04±0.00b
**53**	alpha-acoradiene	C15H24	30.73	1471	1489.77	0.04±0.00a	0.04±0.00a	0.03±0.00ab	0.02±0.00b	0.02±0.00b	0.02±0.00b

**54**	(+)-delta-cadinene	$C_{15}H_{24}$	33.04	1524	1538.15	0.20±0.00cd	0.26±0.08c	0.40±0.03a	0.33±0.01b	0.20±0.02cd	0.16±0.00d
**55**	1,2-Epoxyhexadecane	$C_{16}H_{32}O$	39.5	1708	1730.87	0.21±0.00a	0.16±0.02ab	0.12±0.00b	0.09±0.01b	0.06±0.01c	0.06±0.01c
	**Esters**										
**56**	Methyl salicylate	$C_8H_8O_3$	16.06	1192	1218.12	9.57±1.58e	13.12±0.66d	14.70±0.23cd	16.54±1.53bc	18.53±1.42b	23.93±1.50a
**57**	isopulegyl acetate	$C_{12}H_{20}O_2$	18.41	1259	1261.04	1.74±0.10b	2.11±0.18a	1.59±0.17b	1.45±0.14b	1.21±0.06c	1.18±0.02c
**58**	neryl formate	$C_{11}H_{18}O_2$	19.62	1283	1281.04	21.40±1.63d	23.20±1.90cd	25.10±0.67c	25.55±0.65c	29.23±0.45b	33.05±1.63a
**59**	Geranyl formate	$C_{11}H_{18}O_2$	21.03	1300	1303.00	0.02±0.00c	0.02±0.00c	0.03±0.01b	0.04±0.00ab	0.05±0.00a	0.06±0.01a
**60**	Methyl 2-nonynoate	$C_{10}H_{16}O_2$	21.97	1311	1323.54	0.02±0.00b	0.03±0.00b	0.04±0.00a	0.05±0.00a	0.05±0.01a	0.05±0.01a
**61**	Hex-3-enyl hexanoate	$C_{12}H_{22}O_2$	25.98	1386	1398.29	0.85±0.07e	1.23±0.08d	2.17±0.07c	2.45±0.25bc	2.62±0.18b	3.01±0.10a
**62**	cyclohexyl hexanoate	$C_{12}H_{22}O_2$	26.23	1405	1403.14	0.15±0.00c	0.19±0.01c	0.26±0.03bc	0.23±0.02c	0.33±0.03b	0.49±0.07a
**63**	(z)-8-dodecen-1-yl acetate	$C_{14}H_{26}O_2$	36.3	1605	1608.93	0.04±0.00a	0.02±0.01b	0.02±0.00b	0.01±0.00b	0.01±0.00b	0.01±0.00b
**64**	acrylic acid tetradecyl ester	$C_{17}H_{32}O_2$	41.45	1867	1849.36	0.11±0.01a	0.10±0.01a	0.10±0.00a	0.07±0.01b	0.07±0.00b	0.06±0.00b
	**Terpenes**										
**65**	γ-Terpinene	$C_{10}H_{16}$	9.15	1060	1066.84	1.29±0.14d	1.98±0.16c	3.56±0.27a	2.70±0.07b	2.23±0.18c	1.87±0.11c
**66**	farnesene	$C_{15}H_{24}$	32.50	1508	1526.46	0.02±0.00b	0.02±0.00b	0.03±0.00b	0.04±0.00ab	0.06±0.00b	0.07±0.01a
**67**	trans-Sesquisabinene hydrate	$C_{15}H_{26}O$	35.51	1581	1589.29	1.09±0.09d	1.31±0.08d	2.84±0.54c	3.54±0.27bc	4.27±0.29b	5.44±0.45a
	**Others**										
**68**	Myrtenyl methyl ether	$C_{11}H_{18}O$	13.25	1161	1164.54	0.01±0.00b	0.02±0.00b	0.03±0.00ab	0.03±0.00ab	0.04±0.01b	0.06±0.01a
**69**	Phenol, 3-methyl-	C_7H_8O	9.44	1075	1075.10	1.34±0.08d	1.56±0.03cd	1.61±0.06cd	1.82±0.16c	2.41±0.12b	3.29±0.26a
**70**	n-Amylbenzene	$C_{11}H_{16}$	12.89	1157	1157.28	ND	0.02±0.00b	0.02±0.00b	0.03±0.00ab	0.04±0.00a	0.05±0.00a

**71**	8-Hydroxyquinoline	C9H7NO	23.16	1360	1347.06	1.22±0.09a	1.07±0.06b	0.68±0.04c	0.45±0.03d	0.31±0.06d	0.12±0.00e
**72**	beta-elemene	C15H24	24.77	1391	1377.02	0.02±0.00b	0.03±0.00b	0.03±0.00b	0.04±0.00a	0.05±0.01a	0.05±0.01a
**73**	(+)-Calarene	C15H24	28.06	1432	1440.04	0.07±0.01a	0.04±0.00b	0.03±0.00bc	0.02±0.00c	0.02±0.00c	ND
**74**	2,6-Di-tert-butyl-p-benzoquinone	C14H20O2	30.17	1471	1479.71	ND	ND	ND	0.24±0.03c	0.36±0.02b	0.42±0.02a
**75**	Pentadecanoic acid	C15H30O2	41.89	1867	1880.41	0.47±0.10a	0.38±0.06a	0.26±0.05b	0.23±0.01b	0.21±0.01b	0.17±0.01b
**76**	2-Dodecen-1-yl succinic anhydride	C16H26O3	42.94	1966	1965.02	0.02±0.00b	0.03±0.00b	0.03±0.00b	0.03±0.00b	0.07±0.01a	0.05±0.01a

Note: W0h /CK, W4h, W6h, W8h, W10h, and W12h stand for the withering time set at 0h, 4hrs, 8hrs, 8hrs, 10hrs, and 12hrs, respectively. RT, retention time; ND, not detected; RI, retention index, and RI were determined by normal alkenes (C3-C25). Results were presented as mean ±SD (n=3), and a significant difference is shown by different letters in the same column with a threshold $p<0.05$ according to Duncan's test.

Table S3: Primer sequence used for qRT-PCR

S/N	Enzyme name	Primers	Primer sequence (5'-3')	Amplicon size(bp)
**1**	Isoamylase/debranching enzyme	DBE'- F	TGTACTGAAGGCGGACACTA	123
		DBE'- R	CATCTGAGGCCAGCAATCAT	
**2**	Alpha-amylase-	AMY-F	TCCTGCTGAAACAGTAGTATTCAA	80
		AMY-R	AACTTGAACCACCATGCCCA	
**3**	Beta-amylase	BAM- F	GCTGGGATTCATTGGCACTA	191
		BAM- R	TCTGGTGAGCAGTTTGCATT	
**4**	Dihydroflavonol 4-reductase	DFR-F	AGCATCTCTTGGACTTGCCC	112
		DFR-R	TGGAAGACACCATGACAGCC	
**5**	Anthocyanidin reductase	ANR-F	TCTCACCTCGTAGCACTGGA	182
		ANR-R	ACTCCTTGGATTGCCAGCTT	
**6**	Phosphomevalonate kinase	PVK-F	TAAACCCGACAGCTGGGCAT	188
		PVK-R	CCAATGTTGCAAGGGCTGCT	
**7**	Terpene synthase 10	TPS10-F	ATGGTCCATGCTGCCTCACT	194
		TPS10-R	CGACCGTACCTCCTCAACCG	
**8**	Glutamine synthetase	GLN-F	CTGCCGGAGCGGATAAGTCA	121
		GLN-R	AATTCCCACTGGCCAGGCAT	
**9**	Acyl-CoA oxidase	ACX-F	GTAGCTCTTGGGCGGGAGTT	146

		ACX-R	CCGGTGATTTCCCTGCCTGT	
**10**	Histidinol dehydrogenase	HDH-F	AAGCGGGTAGCAAGGAGCAT	107
		HDH-R	CCAGCAATCTGTGCGGGAAC	
**11**	Hydroperoxide dehydratase	AOS-F	TGGCAAGCAAAGGCAAAGCA	116
		AOS-R	TCCGTCGAGTCCGAGTTTGG	

Table S4: Data Quality for parametric trascriptome sequencing

Sample	raw reads	clean reads	total mapped reads	clean bases	Error rate %	Q20	Q30	GC %
CK-1	46510322	45400296	39132894(86.2%)	6.81G	0.03	96.73	91.16	43.86
CK-2	46501784	45444836	39264299(86.4%)	6.82G	0.03	96.79	91.29	43.77
CK-3	46267538	45304150	39183786(86.49%)	6.8G	0.03	96.9	91.52	43.9
W4h-1	44625170	43732322	37954937(86.79%)	6.56G	0.03	96.93	91.57	43.25
W4h-2	45821170	44000778	38270747(86.98%)	6.6G	0.03	96.66	91.09	44.22
W4h-3	47613696	46484148	40513882(87.16%)	6.97G	0.03	96.9	91.52	43.84
W6h-1	45499730	44197432	38491718(87.09%)	6.63G	0.03	97.07	91.88	43.39
W6h-2	46636792	45488172	39072830(85.9%)	6.82G	0.03	96.84	91.41	43.44
W6h-3	47658026	46169886	40099713(86.85%)	6.93G	0.03	96.75	91.25	42.78
W8h-1	45024722	43334030	37291051(86.05%)	6.5G	0.03	96.73	91.18	44.13
W8h-2	43717878	42535686	36599328(86.04%)	6.38G	0.03	96.87	91.48	43.87
W8h-3	47111512	45714670	39447590(86.29%)	6.86G	0.03	96.72	91.12	43.97
W10h-1	45459654	44359578	38228197(86.18%)	6.65G	0.03	97.02	91.7	43.67
W10h-2	46218924	44969284	38578684(85.79%)	6.75G	0.03	96.73	91.12	43.6
W10h-3	47814898	46274244	40033864(86.51%)	6.94G	0.03	96.99	91.67	43.65

W12h-1	45291374	44206320	38450134(86.98%)	6.63G	0.03	97.1	91.91	43.51
W12h-2	47666044	46658032	40246600(86.26%)	7.0G	0.03	96.63	90.92	43.51
W12h-3	46776080	45832678	39804875(86.85%)	6.87G	0.03	97.02	91.72	43.63

Note: W0h /CK, W4h, W6h, W8h, W10h, and W12h stand for the withering time set at 0h, 4hrs, 8hrs, 8hrs, 10hrs, and 12hrs, respectively.

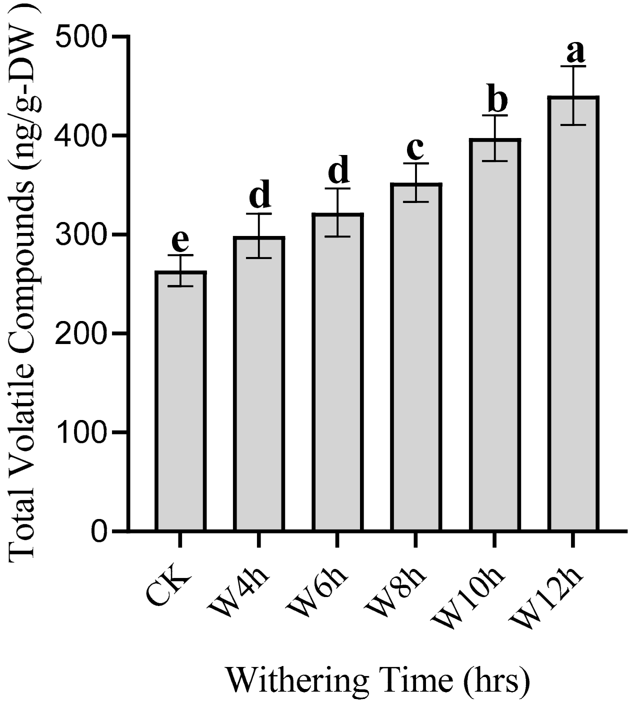

Figure S1: Changes in total volatile compounds during withering process of black tea

Abbreviations of metabolic pathways in Figure 4

PGM, Phosphoglycerate mutase; PSP, phosphoserine phosphatase; TSA1, tryptophan synthase alpha chain; PGDH, D-3-phosphoglycerate dehydrogenase; PSAT, phosphoserine aminotransferase; AK-HSDH_I, aspartate kinase-homoserine dehydrogenase I; HPR, hydroxypyruvate reductase; AGT, alanine-glyoxylate transaminase; SHM, serine hydroxymethyltransferase; TS, threonine synthase; HSK, homoserine kinase; GGT, glutamate:glyoxylate aminotransferase; GK, D-glycerate 3-kinase; AT-IE, phosphoribosyl-AMP cyclohydrolase /phosphoribosyl-ATP pyrophosphohydrolase; APG10, phosphoribosylformimino-5-aminoimidazole carboxamide ribotide isomerase; AT-HF, imidazole glycerol-phosphate synthase; AT-HF, imidazole glycerol-phosphate synthase; HISN6B, histidinol-phosphate aminotransferase; HDH, histidinol dehydrogenase; MEE32, 3-dehydroquinate dehydratase / shikimate

dehydrogenase; SK, shikimate kinase; CM, chorismate mutase; ADH, arogenate dehydrogenase; TSB, tryptophan synthase beta chain; IPDP, 3-isopropylmalate dehydratase protein; IMD, isopropylmalate dehydrogenase; OMR, L-O-methylthreonine resistant; AS, acetolactate synthase I/III small subunit; KARI, ketol-acid reductoisomerase; DAD, dihydroxy-acid dehydratase; BCAT, branched-chain amino acid aminotransferase; MAML, 2-isopropylmalate synthase; GSH1, glutamate-cysteine ligase; GSH2, glutathione synthetase 2; GGT3, gamma-glutamyl transpeptidase 3;LAP, leucyl aminopeptidase; GR, glutathione-disulfide reductase; GGT, glutamate:glyoxylate aminotransferase; ASN, asparagine synthase; PYR B, PYRIMIDINE B; ASPGB, asparaginase / beta-aspartyl-peptidase; OA, omega-amidase; AO, L-aspartate oxidase; ASP4, aspartate aminotransferase 4; ADSS, adenylosuccinate synthase; ASL, adenylosuccinate lyase; GLT, glutamate synthase; GDH, glutamate dehydrogenase; GLN, glutamine synthetase; ASE, midophosphoribosyltransferase; GAD, glutamate decarboxylase; CARB, carbamoyl phosphate synthetase B; OCT, ornithine carbamoyltransferase; NOA, nitric-oxide synthase; SERAT, serine acetyltransferase; CS, cysteine synthase; LCD, L-cysteine desulfhydrase; MS, methionine synthase; MGL, methionine gamma-lyase; SAM, S-adenosylmethionine synthetase; ACS, 1-amino-cyclopropane-1-carboxylate synthase; EFE, ethylene-forming enzyme; AK-HSDH4, aspartate kinase-homoserine dehydrogenase4; ASD, aspartate-semialdehyde dehydrogenase; ALD, LL diaminopimelate aminotransferase; DPE, diaminopimelate epimerase; ARGAH2, arginase
